# Pathology, microbiology, and genetic diversity associated with *Erysipelothrix rhusiopathiae* and novel *Erysipelothrix* spp. infections in southern sea otters (*Enhydra lutris nereis*)

**DOI:** 10.3389/fmicb.2023.1303235

**Published:** 2024-02-01

**Authors:** Ri K. Chang, Melissa A. Miller, Hasan C. Tekedar, Divya Rose, Julio C. García, Benjamin R. LaFrentz, Caitlin E. Older, Geoffrey C. Waldbieser, Eric Pomaranski, Khalid Shahin, Alvin C. Camus, Francesca Batac, Barbara A. Byrne, Michael J. Murray, Matt J. Griffin, Esteban Soto

**Affiliations:** ^1^Department of Medicine and Epidemiology, School of Veterinary Medicine, University of California, Davis, Davis, CA, United States; ^2^Monterey Bay Aquarium, Monterey, CA, United States; ^3^Marine Wildlife Veterinary Care and Research Center, California Department of Fish and Wildlife, Santa Cruz, CA, United States; ^4^Wildlife Health Center, School of Veterinary Medicine, University of California, Davis, Davis, CA, United States; ^5^College of Veterinary Medicine, Mississippi State University, Stoneville, MS, United States; ^6^United States Department of Agriculture, Agricultural Research Service, Aquatic Animal Health Research Unit, Auburn, AL, United States; ^7^USDA-ARS, Warmwater Aquaculture Research Unit, Stoneville, MS, United States; ^8^Aquatic Animal Diseases Laboratory, Aquaculture Division, National Institute of Oceanography and Fisheries, Suez, Egypt; ^9^College of Veterinary Medicine, University of Georgia, Athens, GA, United States; ^10^Department of Pathology, Microbiology, and Immunology, School of Veterinary Medicine, University of California, Davis, Davis, CA, United States

**Keywords:** *Enhydra lutris*, *Erysipelothrix rhusiopathiae*, genetics, pathology, sea otter, septicemia repositories: CP125807, CP125808

## Abstract

*Erysipelothrix* spp., including *E. rhusiopathiae*, are zoonotic bacterial pathogens that can cause morbidity and mortality in mammals, fish, reptiles, birds, and humans. The southern sea otter (SSO; *Enhydra lutris nereis*) is a federally-listed threatened species for which infectious disease is a major cause of mortality. We estimated the frequency of detection of these opportunistic pathogens in dead SSOs, described pathology associated with *Erysipelothrix* infections in SSOs, characterized the genetic diversity and antimicrobial susceptibility of SSO isolates, and evaluated the virulence of two novel *Erysipelothrix* isolates from SSOs using an *in vivo* fish model. From 1998 to 2021 *Erysipelothrix* spp. were isolated from six of >500 necropsied SSOs. *Erysipelothrix* spp. were isolated in pure culture from three cases, while the other three were mixed cultures. Bacterial septicemia was a primary or contributing cause of death in five of the six cases. Other pathology observed included suppurative lymphadenopathy, fibrinosuppurative arteritis with thrombosis and infarction, bilateral uveitis and endophthalmitis, hypopyon, petechia and ecchymoses, mucosal infarction, and suppurative meningoencephalitis and ventriculitis. Short to long slender Gram-positive or Gram-variable bacterial rods were identified within lesions, alone or with other opportunistic bacteria. All six SSO isolates had the *spaA* genotype–four isolates clustered with *spaA E. rhusiopathiae* strains from various terrestrial and marine animal hosts. Two isolates did not cluster with any known *Erysipelothrix* spp.; whole genome sequencing revealed a novel *Erysipelothrix* species and a novel *E. rhusiopathiae* subspecies. We propose the names *Erysipelothrix enhydrae* sp. nov. and *Erysipelothrix rhusiopathiae ohloneorum* ssp. nov. respectively. The type strains are *E. enhydrae* UCD-4322-04 and *E. rhusiopathiae ohloneorum* UCD-4724-06, respectively. Experimental injection of tiger barbs (*Puntigrus tetrazona*) resulted in infection and mortality from the two novel *Erysipelothrix* spp. Antimicrobial susceptibility testing of *Erysipelothrix* isolates from SSOs shows similar susceptibility profiles to isolates from other terrestrial and aquatic animals. This is the first description of the pathology, microbial characteristics, and genetic diversity of *Erysipelothrix* isolates recovered from diseased SSOs. Methods presented here can facilitate case recognition, aid characterization of *Erysipelothrix* isolates, and illustrate assessment of virulence using fish models.

## 1 Introduction

*Erysipelothrix* spp. are Gram-positive or Gram-variable (Dunbar and Clarridge, 2000; Prod’Hom and Bille, 2010; Jean et al., 2019) zoonotic bacterial rods that can cause illness and death in diverse mammals, birds, fish, and reptiles (Wang et al., 2010; Eisenberg et al., 2022). Swine erysipelas, commonly referred to as “diamond skin disease,” caused by *E. rhusiopathiae* is a disease of worldwide economic significance (Opriessnig et al., 2020). The most common form of *Erysipelothrix* spp. in humans is a localized skin infection, erysipeloid, which is often called “seal finger” or “fish handler’s disease”; this condition is an occupational hazard for people who work with infected animals or contaminated soil (Wang et al., 2010). Due to overlapping lesion appearance and terminology, human erysipelas is often confused with swine erysipelas; however, the term “erysipelas” in humans applies to pathology associated with skin infections caused by group A streptococci (Gunderson and Martinello, 2012), while erysipeloid refers to localized *Erysipelothrix* spp. skin infections (Veraldi et al., 2009). Two other forms of *Erysipelothrix* spp.-associated disease in humans are generalized cutaneous pathology and septicemia, often associated with endocarditis (Gorby and Peacock, 1988; Veraldi et al., 2009).

*Erysipelothrix* spp. are ubiquitous and can survive for long periods in the environment, including in the external mucus of fish (Wang et al., 2010; Pomaranski et al., 2018). Novel species within the genus have also been described over the past decade; *E. piscisicarius* is the etiologic agent of piscine erysipelas, a systemic disease in fish, particularly cyprinids, centrarchids, and characids, with recent work suggesting this species may infect a broad range of hosts, including turkeys and swine (Pomaranski et al., 2018, 2020; Grazziotin et al., 2021). In the marine environment, free-living and captive cetaceans are particularly susceptible to morbidity or mortality following *E. rhusiopathiae* infection (Kinsel et al., 1997; Higgins, 2000; Melero et al., 2011). Occasional reports also describe pathology in pinnipeds (Suer et al., 1988; Suer and Vedros, 1988).

In terrestrial mammals, including humans, *Erysipelothrix* spp. infections can manifest as sepsis, dermal lesions, arthritis, endocarditis, and spontaneous abortions (Reboli and Farrar, 1989; Opriessnig et al., 2020). Sheep most commonly exhibit polyarthritis (Opriessnig et al., 2020), while dogs tend to present with septicemia and/or endocarditis (Shimazaki et al., 2005). Birds often have similar lesions to mammals, characterized by acute septicemia, decreased egg production, vegetative valvular endocarditis, and muscle hemorrhage (Opriessnig et al., 2020). Fish infected with *E. piscisicarius* develop necrotizing dermatitis, myositis, and cellulitis, reminiscent of the dermatologic forms observed in terrestrial animals, and the kidney is the most commonly affected visceral organ (Pomaranski et al., 2020). Cetaceans infected with *E. rhusiopathiae* often present with cutaneous vasculitis, similar to swine diamond skin disease, or septicemia, and a mass-mortality event due to *E. rhusiopathiae* was recently documented in harbor porpoises (Suer et al., 1988; Ceccolini et al., 2021; IJsseldijk et al., 2023). Cetacean case reports identify kidney as one of the organs with the greatest concentration of *Erysipelothrix* spp. bacteria, similar to fish (Kinsel et al., 1997). Pinnipeds have been reported to present with cutaneous lesions and lymphadenitis, but also enteritis (Boseret et al., 2012). To our knowledge, no detailed descriptions exist of *Erysipelothrix* spp. infections in southern sea otters (SSO; *Enhydra lutris nereis*).

Laboratory models have also been used to investigate the pathogenesis of *Erysipelothrix* spp. infections and compare virulence of species or strains in different hosts, including swine (To H et al., 2010), mice (Zhu et al., 2018), rats (Renz et al., 1989), chickens (Wattrang et al., 2020), and fish (Pomaranski et al., 2018). *In vivo* models that facilitate study of disease in a controlled environment, such as experimental infection of tiger barbs (*Puntigrus tetrazona*) can facilitate comparison of virulence between *Erysipelothrix* spp. strains (Pomaranski et al., 2018, 2020).

Although *E. rhusiopathiae* was reported anecdotally as a cause of death in SSOs (Miller et al., 2020), a federally-listed threatened species, and one *E. rhusiopathiae* isolate from a SSO has been characterized (Pomaranski et al., 2018), there are no descriptions of *Erysipelothrix* spp.-associated pathology, and little is known about the species and virulence patterns of strains that infect SSOs. The objectives of this study were to estimate the frequency of detection of *Erysipelothrix* spp. in dead SSOs examined by scientists at the California Department of Fish and Wildlife between 1998 and 2021, describe pathology associated with *Erysipelothrix* spp. infection, characterize the genetic diversity and antimicrobial susceptibility of SSO *Erysipelothrix* spp. isolates, characterize novel *Erysipelothrix* species and subspecies, and evaluate the virulence of novel *Erysipelothrix* spp. using an *in vivo* fish model.

## 2 Materials and methods

### 2.1 Necropsy, histopathology, and lesion assessment

Case records for all minimally decomposed, necropsied SSOs that were culture-positive for *Erysipelothrix* spp. from 1998 through 2021 were assessed for possible bacterial-associated pathology, following a standardized protocol that included microscopic examination of all major tissues (Miller et al., 2020). *Erysipelothrix* spp. infection was considered a cause of death or sequela by a veterinary pathologist based on review of all findings from gross necropsy, microbiology, and histopathology, including any other concurrent health conditions. Factors that were considered in this assessment included bacterial isolation from observed lesions and visualization of morphologically compatible bacteria (Gram-positive or Gram-variable, short or long, filamentous bacterial rods) within lesions on histopathology. Variable (Gram-positive, Gram-negative, or metachromatic) staining for *Erysipelothrix* spp. has been reported in other studies (Dunbar and Clarridge, 2000; Prod’Hom and Bille, 2010; Jean et al., 2019). *Erysipelothrix* spp. bacterial morphology can also vary from short to long, slender, even filamentous rods (Reboli and Farrar, 1989; Dunbar and Clarridge, 2000; McLauchlin and Riegel, 2012).

Lesions that appeared to be associated with *Erysipelothrix* spp. infection were summarized based on findings from necropsy, histopathology, bacterial culture, and Gram stains. Although individual case detail varied, all available clinical, gross necropsy, histopathology, and diagnostic data were reviewed to assess potential *Erysipelothrix* spp.-associated lesion patterns. Archival records were also reviewed to determine the proportion of live-sampled SSOs that were *Erysipelothrix* spp.-positive and identify any culture-positive sample locations.

### 2.2 Bacterial isolation and identification

Sterile swabs inoculated from body fluids or tissue during necropsy were shipped overnight to the Microbiology Laboratory of the University of California-Davis Veterinary Medical Teaching Hospital for bacterial isolation and identification. Subsamples of all major tissues and body fluids were also cryopreserved at −80°C. Bacteriological swabs were inoculated onto sheep blood agar ((SBA), tryptic soy agar supplemented with 5% sheep blood) (Hardy Diagnostics) and MacConkey agar (Hardy Diagnostics), and incubated at 35°C in 5% CO_2_ for 24–72 h. Purified isolates were identified using conventional microbiological methods, including colony morphology, Gram stain, catalase test, and reaction on triple sugar iron agar (Biological Media Services (BMS)). Isolates cultured after 2013 had identity confirmed via matrix-assisted time-of-flight mass spectrometry (MALDI-TOF) (Biotyper, Bruker) using the extended direct transfer method. Isolates were stored on ProLab Microbank Beads (ThermoFisher Scientific) at −80°C for further study. Cryopreserved isolates were first plated on SBA (BMS), then colonies were swabbed and inoculated into brain heart infusion broth (Sigma-Aldrich) and incubated at 28°C at 150 RPM overnight. *Erysipelothrix* spp. identity was confirmed via MALDI-TOF for cryopreserved isolates.

For any necropsied SSO with a previous *Erysipelothrix* spp. isolate that had not been cryopreserved, necropsy tissues cryoarchived at −80°C were thawed, and fresh bacterial swabs were collected and submitted for isolation. Prior sources of *Erysipelothrix* spp. isolation from necropsied SSOs (lymph nodes, kidney, spleen, liver, lung, brain, and eye) were prioritized for re-isolation attempts. Archival tissues were thawed, and a fresh bacterial swab was collected, streaked on blood agar plates, and incubated at 30°C for 5 days. During incubation, any visible bacterial colonies were inspected for morphology consistent with *Erysipelothrix* spp. and subcultured onto fresh media to isolate single strains. Suspect colonies were Gram stained, morphologically identified, and bacterial identity was confirmed via MALDI-TOF.

### 2.3 Antimicrobial susceptibility testing

The minimum inhibitory concentrations for 18 different antimicrobials were identified using Sensititre Avian AVIAN1F Vet AST plates (ThermoFisher Scientific) in cation-adjusted Mueller Hinton broth supplemented with lysed horse blood (ThermoFisher Scientific) following CLSI protocols (CLSI, 2017) for five of the six SSO isolates. The sixth SSO isolate was not cryopreserved and was not included in this analysis. *Streptococcus pneumoniae* ATCC® 49619 was used as a quality control. Plates were incubated at 37°C for 22 h.

### 2.4 Characterization of surface protective antigen

DNA was extracted from confirmed *Erysipelothrix* spp. by presence of a *spa* gene using a DNeasy Blood and Tissue Kit (QIAGEN) following manufacturer protocols. All DNA samples were stored at −20°C until used. The *spa* type of each isolate was determined using a previously established multiplex PCR (Shen et al., 2010; Supplementary Table S1) with the modification of using a Multiplex PCR Kit (QIAGEN) (25 μL Master Mix, 5 μL 10x primers, 15 μL nuclease-free water, 5 μL extracted DNA to a final volume of 50 μL).

### 2.5 Multi-locus sequence analysis

Eight housekeeping genes were amplified using Phusion Hot Start II High Fidelity DNA Polymerase following manufacturer protocols (ThermoFisher Scientific): *gpsA* (glycerol-3-phosphate-dehydrogenase), *recA* (recombinase A), *purA* (adenylosuccinate synthetase), *pta* (phosphate acetyltransferase), *prsA* (ribose-phosphate pyrophosphokinase), *galK* (galatokinase), and *ldhA* (D-lactate dehydrogenase), and *gyrB* (gyrase B) based on a scheme by Pomaranski et al. (2020). Thermocycling protocol included 30 s denaturation at 98°C, followed by 30 cycles of denaturation at 98°C for 10 s, annealing at 55°C for 20 s, and extension at 72°C for 50 s, and a final extension at 72°C for 6 min. The PCR was performed using a SimpleAmp Thermal Cycler Applied-Biosystem (ThermoFisher Scientific). Primer sequences are summarized in Supplementary Table S2. PCR products were purified using QIAquick PCR Purification Kit (QIAGEN) following manufacturer protocols and submitted for sequencing using the forward primer. Sequencing was performed by GENEWIZ.

Contiguous sequences were assembled using MEGA v.7 (Tamura and Nei, 1993; Kumar et al., 2016). Sequences were concatenated in Geneious 10.2.4 (Kearse et al., 2012) then aligned with MUSCLE (Edgar, 2004) and the final tree was constructed using 1,000 bootstrap replicates and maximum-likelihood analysis and Tamura-Nei model. The final tree included SSOs (*n* = 6) and previously *spa*-typed isolates from fish (*n* = 20), marine mammals (*n* = 7), terrestrial mammals (*n* = 6), and birds (*n* = 1; Supplementary Table S3; Pomaranski et al., 2018).

### 2.6 Whole genome sequencing of novel *Erysipelothrix* spp.

*Erysipelothrix* spp. isolates 4322–04 and 4724–06 from SSOs were sequenced using both short read (Illumina) and long-read [Oxford Nanopore Technologies (ONT)] approaches. Illumina sequencing was performed by Azenta Life Sciences in a Hiseq-4000. Nanopore sequencing was performed on a GridION platform (Oxford Nanopore Technologies plc) employing the ligation sequencing kit (LSK109) and R9.4.1 flow cells. Nanopore reads were filtered to a minimal quality score of 13 and a minimal length of 6,000 bp using NanoFilt v2.2.0 (De Coster et al., 2018), then long reads from each isolate were assembled with Canu v1.8 (Koren et al., 2017) using default parameters and declaring an estimated genome size of 1.7–1.8 Mb. Resulting genomes were then polished with Medaka v1.4.3. Illumina reads were quality filtered with trimmomatic v0.39 (Bolger et al., 2014; LEADING:25 TRAILING:25 SLIDINGWINDOW:4:25 MINLEN:50 ILLUMINACLIP:NexteraPE-PE.fa:2:30:10:2:True) before being used to polish ONT assemblies with two rounds of pilon v.1.24 (Walker et al., 2014), using minimap2 (v2.24-r1122; Li, 2021) and samtools (v1.15.1; Danecek et al., 2021). The sequenced genomes were initially annotated using the RAST server (Rapid Annotations using Subsystems Technology) with the Glimmer option (Salzberg et al., 1998; Aziz et al., 2008). Genomes were also annotated employing the National Centre for Biotechnology Information’s (NCBI) Prokaryotic Genome Annotation Pipeline (PGAP) for submission to GenBank (Tatusova et al., 2016).

Taxonomic affiliations of the study isolates were determined based on inferences from Average Nucleotide Identity (ANI)^1^ and digital DNA–DNA hybridization (dDDH)^2^ assessments (Konstantinidis and Tiedje, 2005a,b; Goris et al., 2007; Richter and Rosselló-Móra, 2009; Auch et al., 2010; Meier-Kolthoff et al., 2013). Comparisons were made against 33 discrete *Erysipelothrix* spp. genomes downloaded from NCBI’s Reference Sequence Databank (RefSeq). ANI values were calculated using FastANI v1.33 (Jain et al., 2018), while dDDH estimations were conducted using the Leibniz Institute’s Genome-to-Genome Distance Calculator (GGDC v3.0) employing the BLAST+ option (Meier-Kolthoff et al., 2021). All downloaded RefSeq genomes were annotated by NCBI’s PGAP. Orthologous gene sets of the core genome were identified using Edgar 2.0, and individually aligned using MUSCLE (Edgar, 2004). The resulting alignments were concatenated, and phylogenetic distances were calculated based on the complete core genome using the neighbor-joining method in PHYLIP (Baum, 1989). The phylogenetic tree was built out of a core of 428 genes present in all 35 genomes included in the analysis, resulting in 14,980 genes in total. Further, all 16S rRNA copies from *Erysipelothrix* spp. 4322–06 (*n* = 6), 4724–06 (*n* = 7) and other *Erysipelothrix* spp. genomes were obtained using localized Blastn searches in Geneious Prime and aligned using MAFFT (Katoh et al., 2009). Lastly, amino acid sequences of putative *spa* genes for isolates 4322–04 and 4724–06 were aligned (ClustalW; MEGA v.7) with representative sequences from 16 different *Erysipelothrix* spp. isolates representing *spaA*, *spaB,* and *spaC*-type strains (To and Nagai, 2007).

### 2.7 Fatty acid and biochemical characterization

Biochemical characterization of five *E. rhusiopathiae* isolates; three *spaA* (6567, H1T1, 7692–15), two *spaB* (7122, 7155), one *E. piscisicarius* isolate (15TAL064) and two novel *Erysipelothrix* spp. isolates from SSOs (4322–04 and 4724–06) was performed using Rapid ID 32 STREP (BioMérieux) according to manufacturer instructions with minor modifications. Triplicate SBA plates for each of the eight *Erysipelothrix* isolates were inoculated and incubated for 5 d at 30°C. Cells were harvested from the SBA plates using sterile swabs and inoculated into 2 mL API® suspension medium and the turbidity was adjusted to 4 McFarland density. The suspension medium was immediately used to inoculate each strip by dispensing 55 μL into each biochemical reaction cupule. The strips were then incubated for 4.5 h at 36.5°C. Each strip was read visually, and results were interpreted using the *mini* API identification system (BioMérieux). The presence of oxidase and catalase enzyme for each isolate was also determined. For the oxidase test, a single colony was selected from individual SBA plates and streaked onto a grade 1 85 mm filter paper (Whatman), then a single drop of Remel™, BactiDrop™, Oxidase (Lenexa) was added to the colony. Catalase tests were prepared by harvesting a small amount of bacterial culture from SBA and streaking onto a clean microscope slide. Then, one drop of 30% hydrogen peroxide solution was added to the slides.

Five *E. rhusiopathiae* isolates (6567, 7122, 7155, H1T1 and 7692–15), one *E. piscisicarius* isolate (15TALS064) and two novel *Erysipelothrix* spp. isolates (4322–04 and 4724–06) were included in the fatty acid methyl ester (FAME) analysis and this assay was conducted as previously described for *E. piscisicarius* (Pomaranski et al., 2020). Following gas chromatography, samples were analyzed using the Sherlock Microbial Identification System (MIS) RCLIN6 6.2 library (version 6.2. MIDI, Inc.). The triplicate FAME results for each isolate were averaged and results reported.

### 2.8 Tiger barb *in vivo* challenges

*In vivo,* intracoelomic bacterial challenges using the novel *Erysipelothrix* sp. isolates 4322–04 and 4724–06 and an *E. piscisicarius* isolate were conducted in tiger barbs (TBs). Archived isolates were revived from cryogenic storage on SBA (72 h at 30°C) and colonies resuspended in phosphate buffer saline (PBS) to an estimated concentration of 10^8^ CFU/mL. Laboratory-controlled challenges were performed at the Center for Aquatic Biology and Aquaculture, University of California-Davis, CA in compliance with the University of California-Davis Institutional Animal Use and Care Committee. Naïve TBs were kindly donated by Dr. Roy Yanong from the Tropical Aquaculture Laboratory (Ruskin, FL) and acclimated in 130 L tanks holding ~90 L of fresh well water at 22 ± 2°C under flow through conditions with constant aeration 1 week prior to challenge and fed commercial feed (Tetramin Tropical Flake Feed, Tetra) at 1% body weight daily.

The day of challenge, TBs were anesthetized using 100 mg/L of sodium bicarbonate buffered MS-222 (tricaine methanesulfonate, Western Chemical, Inc). Each fish was injected intracelomically with 50 μL of a 10^7^ CFU/fish bacterial suspension or PBS (n = 15/treatment) and added to 19 L tanks with the same conditions they were originally acclimated in (n = 15 fish/tank). Fish were monitored twice daily for 21 days for morbidity and mortality. Fresh dead fish were collected, and brains plated on SBA then fixed in 10% buffered formalin for histological examination. Moribund fish were euthanized using 500 mg/L sodium bicarbonate buffered MS-222 and similarly plated and fixed. Survivors were euthanized 21 days after exposure using the above protocol and three fish/tank fixed in 10% buffered formalin for histological examination with the remaining fish having their brains plated.

Six fish (three mortalities and three 21-day survivors from each of the two challenge groups), and five control fish were examined histologically. Whole formalin-fixed fish were decalcified for 12 h in Kristensen’s solution, serially sectioned transversely, and the sections placed into tissue cassettes. Tissues were processed routinely by dehydrating in a series of ethanol solutions, clearing in xylenes, embedding in paraffin, and sectioning at 5 μm. All prepared sections were stained with hematoxylin and eosin (H&E). Select sections were stained with a modified Brown and Hopps tissue Gram stain (B&H).

Small, spherical, γ-hemolytic colonies from brain plating consistent with *Erysipelothrix* spp. were inoculated in brain heart infusion broth and incubated at 30°C and rotated at 110 RPM. DNA was extracted from pelleted culture aliquots using the DNeasy Blood and Tissue Kit (QIAGEN) following manufacturer protocols. *GyrB* was used for PCR confirmation of *Erysipelothrix* sp. identity using the same protocol as MLSA sequencing.

## 3 Results

### 3.1 Findings from gross necropsy, histopathology, special stains, and bacterial culture

Of approximately 500 SSOs necropsied at the Marine Wildlife Veterinary Care and Research Center from 1998 to 2021 with samples submitted for microbial testing, six were culture positive for *Erysipelothrix* spp. (Table 1). Biochemical and MALDI-TOF characterization of the six isolates from necropsied SSOs determined that four of the isolates were *E. rhusiopathiae* and two were *Erysipelothrix* spp. strains, with no apparent temporal or spatial clustering in relation to bacterial species (Table 1).

**Table 1:** *Erysipelothrix* spp. isolates recovered from necropsied southern sea otters (*Enhydra lutris nereis*) used in this study.

Isolate ID	Bacterial species	Bacterial source	Type of culture	Primary cause of death	Strand date	Sex/age class	Animal stranding location	Full histology
3111–98	*Erysipelothrix rhusiopathiae*	Lung	Mixed	Suspect bacterial pneumonia	12/1998	M/Pup	Pacific Grove, CA	No
4322–04	*Erysipelothrix enhydrae* sp. nov.	R axillary LN, R pre-scapular LN	Pure (LN)	Bacterial sepsis	9/2004	M/Adult	Santa Cruz, CA	Yes
4724–06	*Erysipelothrix rhusiopathiae ohloneorum* ssp. nov.	Kidney, eye, R retropharyngeal LN	Pure (eye, LN)	Bacterial sepsis	4/2006	F/Subadult	Carmel-By-The-Sea, CA	Yes
5818–10	*E. rhusiopathiae*	L inguinal LN, spleen, R axillary LN, transmitter surface	Pure (all sites)	Bacterial sepsis	6/2010	F/Adult	Monterey, CA	Yes
6640–12	*E. rhusiopathiae*	L axillary LN, kidney infarct, kidney pelvis	Pure (all sites)	Bacterial sepsis	11/2012	M/Aged adult	Moss Landing, CA	Yes
7692–15	*E. rhusiopathiae*	Lung, feces	Mixed	Segmental enterocolitis	10/2015	F/Adult	Monterey, CA	Yes

LN, lymph node. Bacterial identity was confirmed via matrix-assisted time-of-flight mass spectrometry (Biotyper, Bruker).

The six SSOs that were culture-positive for *Erysipelothrix* spp. included four adults or aged adults, one pup, and one subadult, and equal numbers of males and females. Infected SSOs stranded during various calendar months. Although the SSO geographic range averaged around 500 km during the study period, all six *Erysipelothrix* spp.-positive animals stranded within 85 km of each other within a coastal region centered on Monterey Bay in central California. *Erysipelothrix* spp. were commonly isolated from lymph nodes (*n* = 4 animals, *n* = 6 total lymph nodes), kidney (*n* = 2), and lung (*n* = 2), but also from feces, spleen, and aqueous humor fluid from the eye. *Erysipelothrix* spp. were isolated in pure culture from all sample locations for half of cases (3/6); pure cultures were commonly obtained from lymph nodes (*n* = 4/6), and kidney (*n* = 2/2). For 13 total isolations of *Erysipelothrix* spp. across culture swabs from six SSOs, the bacterium was isolated in pure culture in 77% of samples submitted (*n* = 10/13), and as mixed growth with other opportunistic bacterial pathogens from three samples. Mixed bacterial growth was associated with swabs collected from feces, frozen–thawed lung, and a lymph node (Table 1). Other known SSO bacterial pathogens that were co-isolated with *Erysipelothrix* spp. included *Streptococcus phocae* and *Vibrio parahemolyticus* (Miller et al., 2020).

All six *Erysipelothrix* spp.-positive SSOs were thin or emaciated. Although lung from SSO 3111–98 was culture-positive for *E. rhusiopathiae* and pneumonia was suspected as the primary cause of death, limited histopathology sampling, freeze–thaw artifact and moderate autolysis precluded definitive diagnosis. As a result, assessment of gross and microscopic lesions in relation to bacterial detection was limited to the remaining five cases.

#### 3.1.1 SSO 4322–04

**Figure 1:**
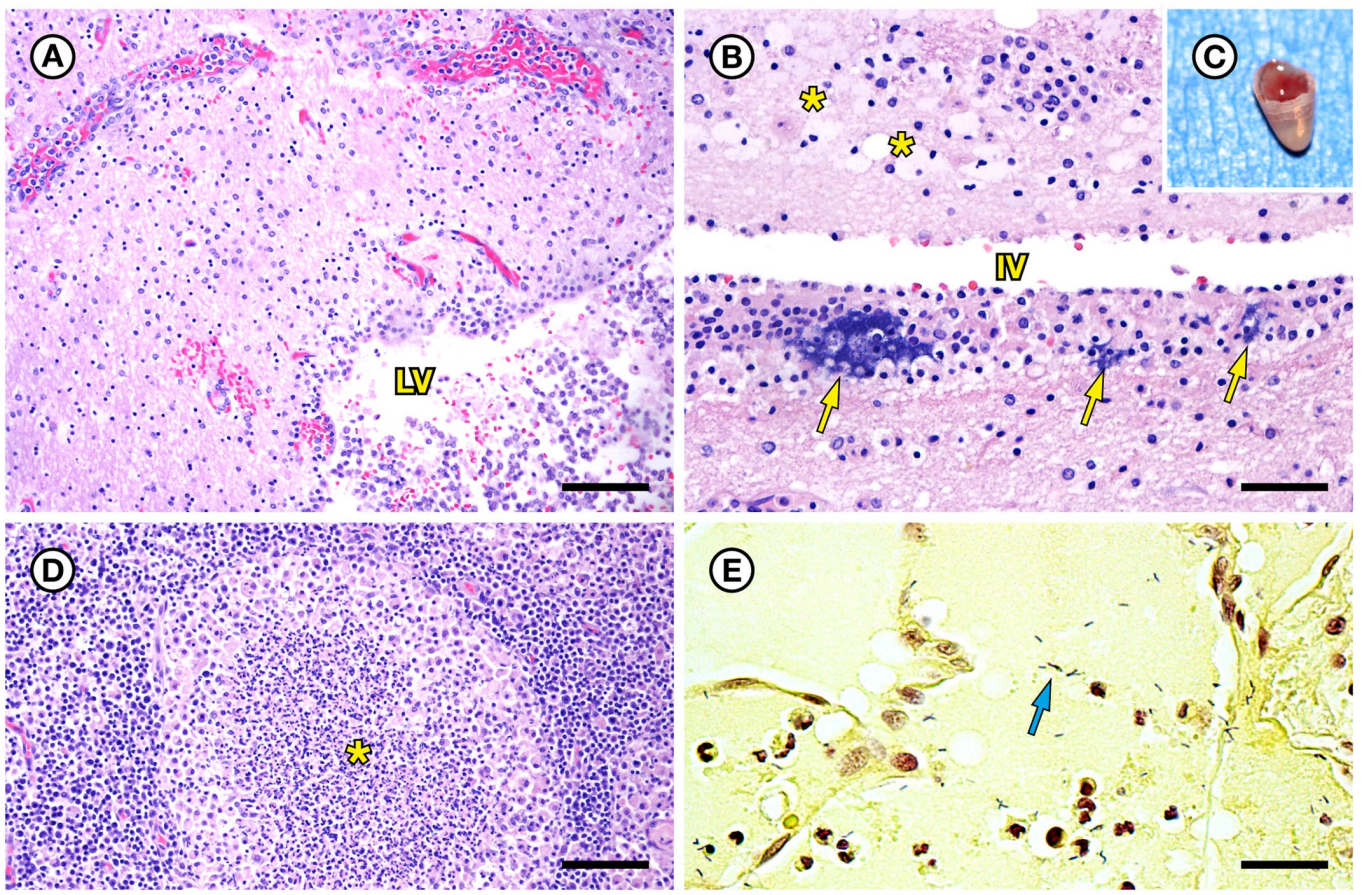
Histopathologic findings from southern sea otter (*Enhydra lutris nereis*) 4322–04 (*E. enhydrae* sp. nov.). **(A)** Cerebrum and lateral ventricle (LV): There is severe suppurative encephalitis and ventriculitis with multifocal hemorrhage (H&E; Bar = 95 μm). **(B)** Higher magnification view of brain tissue around fourth ventricle (IV), showing suppurative inflammation, hemorrhage, periventricular astrogliosis (asterisks) and intralesional masses of bacteria, dominated by long, slender bacilli (arrows). Gram stains revealed a mixture of Gram-positive and Gram-negative bacteria of varying morphology, including numerous long, slender Gram-positive bacilli within and adjacent to the inflamed ventricles (H&E; Bar = 50 μm). **(C)** Confirmed intraspecific fight trauma: The partially degraded tip of a sea otter canine tooth was embedded within a mass of scar tissue in the subcutaneous fascia of the right flipper (gross). **(D)** Large mass of neutrophils (asterisk) within the medullary sinus of an inflamed axillary lymph node (H&E; Bar = 90 μm). **(E)** Gram stain of blood vessels adjacent to the axillary lymph node shown in D. Numerous small Gram-positive bacterial rods are visible within the vascular lumen (arrow)(Gram; Bar = 50 μm).

**Figure 2:**
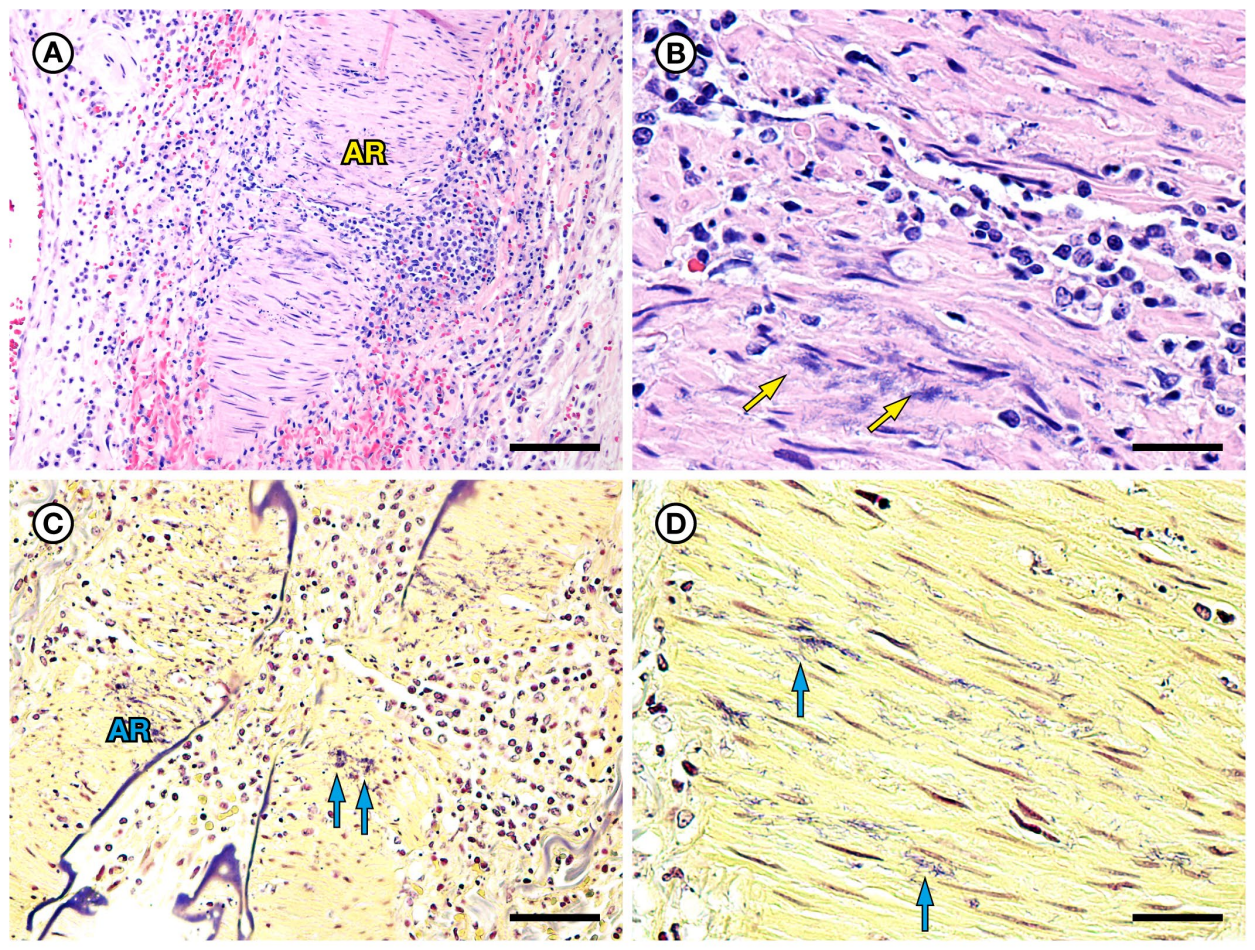
Histopathologic findings from southern sea otter (*Enhydra lutris nereis*) 4322–04 (*E. enhydrae* sp. nov.) **(A)** Tangential section of perirenal artery (AR) showing suppurative and lymphohistiocytic arteritis and peri-arteritis with perivascular hemorrhage (H&E; Bar = 95 μm). **(B)** Higher magnification view of the same artery **(A)**: Clusters of slender bacterial rods (arrows) are visible within the tunica media, associated with a mixed inflammatory infiltrate dominated by neutrophils (H&E; Bar = 25 μm). **(C)** Gram stain of the same inflamed artery (AR). Numerous Gram-positive bacteria are visible within the tunica media (arrows) (Gram; Bar = 95 μm). **(D)** Higher magnification view of the artery shown in **C**; thousands of slender Gram-positive bacterial rods are visible within the tunica media of the inflamed artery (arrows) (Gram; Bar = 25 μm).

SSO 4322–04 was an adult male with gross evidence of repeated intraspecific trauma suggestive of territorial fights between males, as indicated by the tip of a sea otter canine tooth recovered from a chronic fascial wound on the right flipper (Figure 1C). Histopathology revealed mixed bacterial infection of the brain and ventricles (Figures 1A,B), and severe multifocal suppurative lymphadenitis (Figure 1D). Gram stains of the axillary lymph node revealed small numbers of long, slender Gram-positive bacterial rods within pericapsular blood vessels and the subcapsular sinus (Figure 1E). Similar long, slender Gram-positive bacilli were also visible in the brain, admixed with smaller Gram-negative rods and Gram-positive cocci. Also noted was moderate to severe multifocal fibrinohemorrhagic and suppurative mural arteritis in a renal artery, associated with numerous intramural slender, filamentous bacilli within the tunica media (Figures 2A,B). Special stains confirmed the vascular mural bacilli as Gram-positive (Figures 2C,D).

Bacterial culture yielded pure growth of *Erysipelothrix* sp. from the right prescapular lymph node, and a mixture of *Erysipelothrix* sp. and *S. phocae* in the right axillary lymph node (Table 1). *Streptococcus phocae* and *V. alginolyticus* were also isolated from heart blood and lung. The primary cause of death was bacterial septicemia, characterized by multifocal suppurative lymphadenitis, arteritis, meningoencephalitis, ependymitis and ventriculitis, with multifocal hemorrhage, occasional intravascular fibrin thrombi, vascular fibrinoid necrosis and large numbers of free and intracytoplasmic long, slender bacterial rods in multiple tissues. Based on cumulative results from gross necropsy, histopathology, culture, and special stains, *Erysipelothrix* sp. was considered the major opportunistic pathogen, with contributions by *S. phocae* and *V. alginolyticus*. Additional causes of morbidity and mortality for this SSO included recurrent fight trauma, gastric ulcers, cardiomyopathy, emaciation, and chronic dental disease. Three potential portals of bacterial invasion were considered in this case: recurrent fight trauma, gastric ulcers, and dental disease. Although fight wounds were considered the most likely source given the gross lesions and isolation of *S. phocae*, a confirmed SSO bacterial pathogen significantly associated with external wounds in SSOs (Bartlett et al., 2016), this could not be proven.

#### 3.1.2 SSO 4724–06

**Figure 3:**
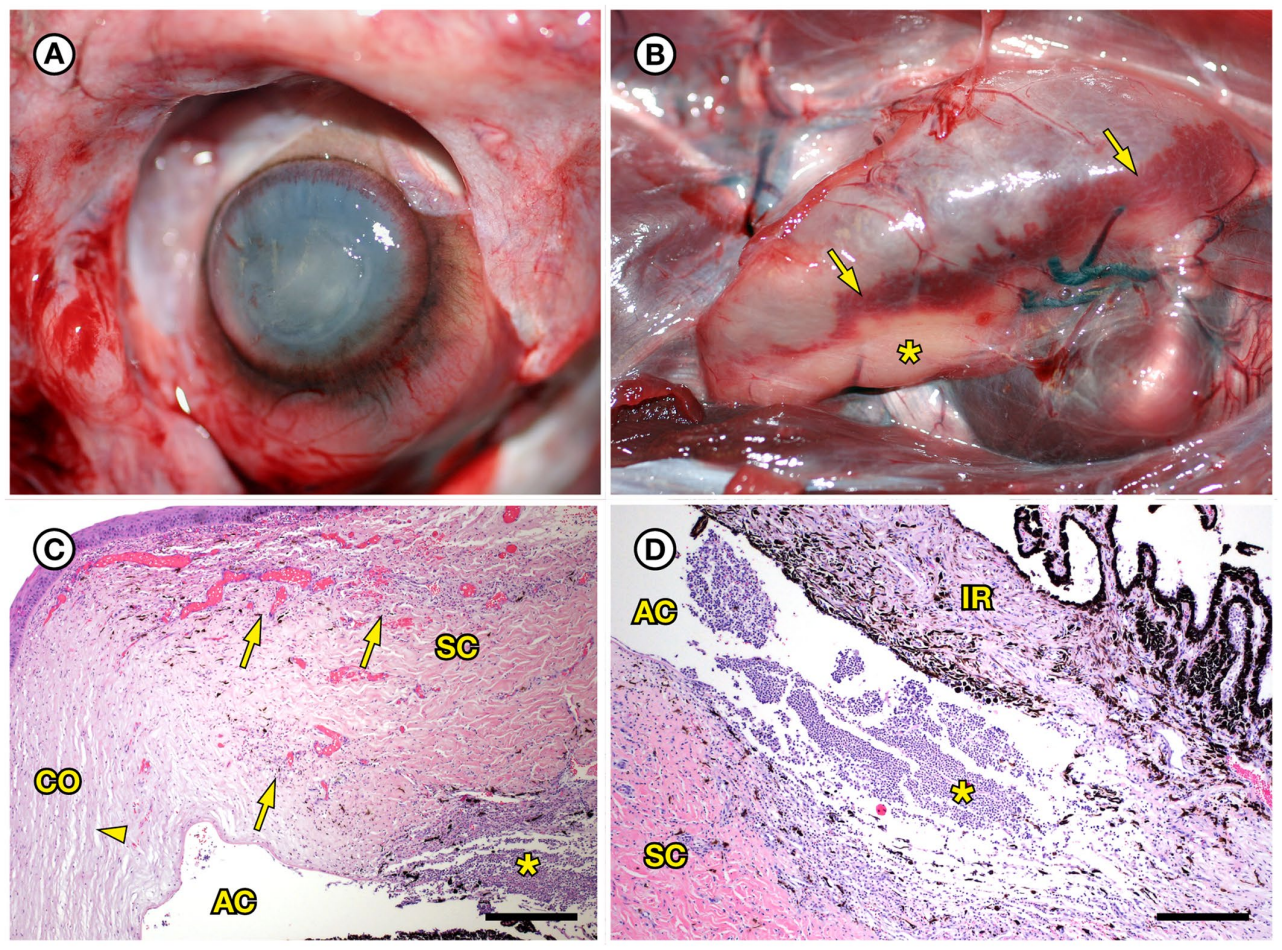
Gross and histopathologic findings from southern sea otter (*Enhydra lutris nereis*) 4724–06 (*E. rhusiopathiae ohloneorum* ssp. nov.). **(A)** Eye with severe corneal edema, scleral injection and suspected endophthalmitis and hypopyon. **(B)** All lymph nodes of the head and neck, including the retropharyngeal lymph node shown here were enlarged, solid, and hemorrhagic; hemorrhage flowing into the lymph node from adjacent tissue and lymphatics is apparent as an irregular bright red line along the bottom edge of the lymph node (arrows). The broad expanse of tan tissue within the lymph node (asterisk) suggests a concurrent influx of inflammatory cells. **(C)** Junction of cornea (CO), sclera (SC) and anterior chamber (AC) of the eye shown in **A**: There is scleral congestion, hemorrhage and suppurative inflammation (arrows). The iridocorneal angle of the anterior chamber, just visible at bottom right, contains a dense infiltrate of neutrophils admixed with sparse red blood cells (asterisk). There is also moderate corneal edema (arrowhead) (H&E; Bar = 220 μm). **(D)** Higher magnification view of the iridocorneal angle of the anterior chamber, showing high numbers of neutrophils (asterisk) in the anterior chamber (AC), confirming the gross diagnosis of endophthalmitis and hypopyon. The locations of the sclera (SC) and iris (IR) are shown for reference (H&E; Bar = 220 μm). Although no bacteria were detected in Gram stains of the eye, *E. rhusiopathiae ohloneorum* ssp. Nov. was isolated in pure culture from anterior chamber fluid.

SSO 4724–06 was a subadult female found alive, obtunded, and dyspneic with severe muscle fasciculations, hypothermia, and hypoglycemia and was euthanized without treatment due to a poor prognosis. Gross necropsy and histopathology revealed severe bilateral suppurative anterior uveitis, scleritis, keratitis and endophthalmitis with hypopyon (Figures 3A,C,D), likely resulting in blindness. All cephalic, cervical, and axillary lymph nodes were enlarged, meaty, and hemorrhagic (Figure 3B), and moderate suppurative lymphadenitis was confirmed on histopathology. Although no bacteria were detected in a Gram stain of an inflamed eye, swabs of aqueous humor fluid from the eye and the right retropharyngeal lymph node yielded pure growth of *Erysipelothrix* sp. Based on findings from gross necropsy, histopathology, and bacterial culture, the primary cause of death was bacterial sepsis due to *Erysipelothrix* sp. infection. Contributing causes of morbidity and mortality included mild acanthocephalan peritonitis, gastric ulcers, emaciation, and mild multifocal meningoencephalitis, myocarditis and myositis associated with intralesional *Toxoplasma gondii* protozoal parasites. Potential portals of bacterial invasion include acanthocephalan peritonitis and gastric ulcers.

#### 3.1.3 SSO 5818–10

SSO 5818–10 was a tagged adult female that had a very high frequency (VHF) transmitter and core body temperature and time depth recorder (TDR) that had been surgically placed intraperitoneally 4 months prior to death. During the 2 weeks prior to death, field monitoring revealed potential copulatory behavior and more time spent hauled out of water than usual. Data collected the night before death revealed a significant drop in core body temperature, from 39.2°C after entering the water from a haul-out due to human disturbance, to 36.9°C when she returned to the same haul-out. She was emaciated with a saturated pelage, and she appeared to be unable to maintain her core body temperature in water.

Gross necropsy revealed a moderate nose wound (indicative of recent copulatory activity), systemic lymphadenopathy, gastric ulcers, and multifocal splenic infarcts. Based on partial atrophy of her mammary glands, the presence of a recent nose wound, emaciation, and identification of a large corpus luteum on her left ovary, end-lactation syndrome (ELS) (Chinn et al., 2016) was considered a contributing cause of death.

**Figure 4:**
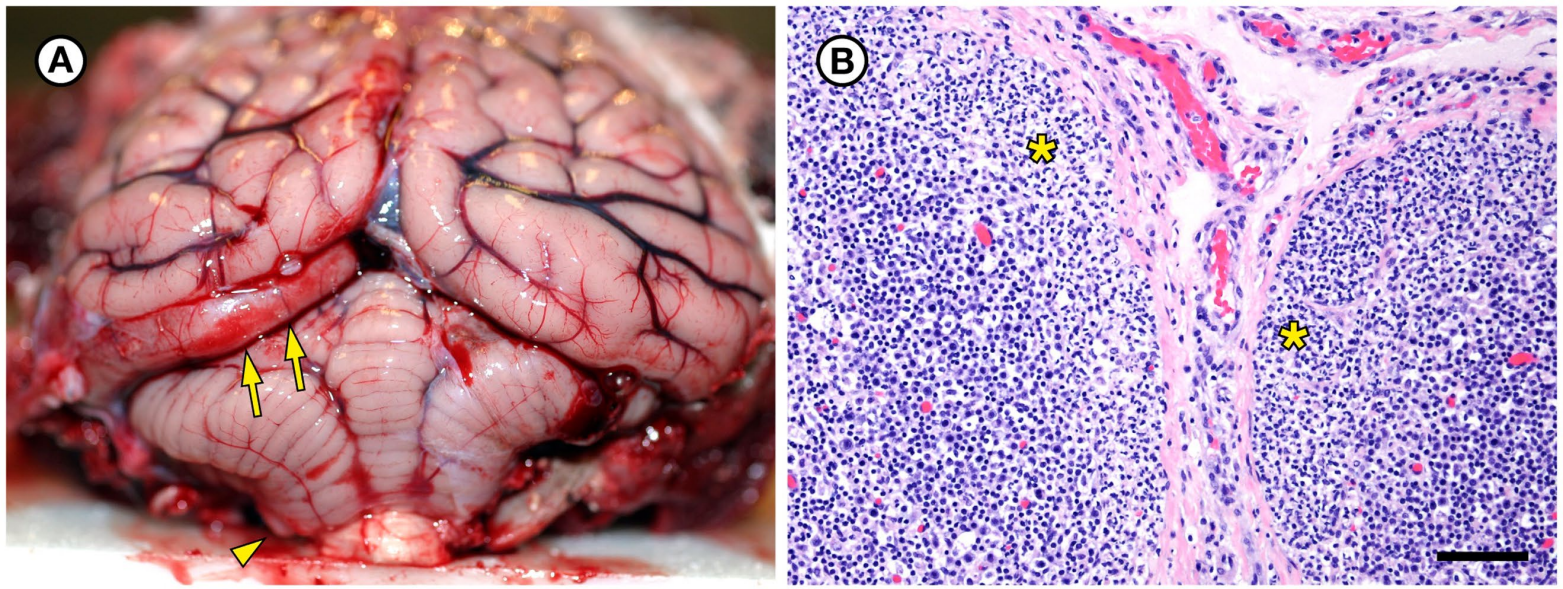
Gross and histopathologic findings from southern sea otter (*Enhydra lutris nereis*) 5818–10. **(A)** The brain was moderately and diffusely swollen, resulting in focally severe subtentorial herniation of the left occipital lobe (arrows). Mild coning and superficial hemorrhage of the caudal cerebellar vermis is also visible (arrowhead). Both lesions are indicative of diffuse, severe brain swelling. Histopathology revealed severe brain congestion and perivascular to diffuse edema, likely secondary to acute sepsis-associated vasculopathy. **(B)** Superficial cortex and capsule of axillary lymph node from the same sea otter, showing marked expansion of the subcapsular sinuses by neutrophils (asterisks). *Erysipelothrix rhusiopathiae* was isolated in pure culture from this lymph node and three other locations at gross necropsy. No other bacteria were isolated from any of the four culture sites (H&E; Bar = 85 μm).

Also noted at necropsy was severe diffuse brain swelling with acute sub-tentorial herniation of her left cerebral occipital lobe (Figure 4A). There was marked diffuse dilation and congestion of subcutaneous veins. Brain histopathology revealed marked, diffuse brain and meningeal congestion and marked edema, which was especially prominent in the perivascular neuropil and meninges. In some areas this was accompanied by mild perivascular hemorrhage. Intravascular red blood cells appeared to be partially hemolyzed in some areas of the brain. The cardiac musculature was also diffusely congested, but inflammation was relatively mild in both tissues and was composed of a mixture of lymphocytes, macrophages, and sparse neutrophils. Superimposed on the acute, severe brain congestion and edema were microscopic lesions in the heart, hippocampus, and thalamus suggestive of prior (subacute to chronic) sublethal domoic acid intoxication, including mild to moderate segmental loss of hippocampal pyramidal neurons in the CA2 sector (Miller et al., 2021). Postmortem urine was trace-positive for domoic acid (0.9 ng/g), a neurotoxin produced by harmful algal blooms by *Pseudo-nitzchia australis*, via ELISA.

Histopathology of the enlarged, reactive lymph nodes revealed moderate to severe diffuse suppurative lymphadenitis (Figure 4B). *Erysipelothrix* sp. was isolated in pure culture from the right axillary and left inguinal lymph nodes, spleen, and the surface of the intraperitoneal VHF transmitter; no other bacteria were isolated from samples collected at necropsy (Table 1). The collective findings from gross necropsy, histopathology, and bacterial culture were supportive of acute bacterial septicemia as the primary cause of death. Other conditions that could have contributed to morbidity and mortality included end lactation syndrome (Chinn et al., 2016), possible prior sublethal domoic acid intoxication, gastric ulcers, dehydration, and terminal seawater aspiration (drowning). Potential portals of bacterial invasion include the nose wound and gastric ulcers. Isolation of *Erysipelothrix* spp. from the VHF transmitter surface was considered a spurious result in a septicemic animal, reflective of systemic bacterial infection.

#### 3.1.4 SSO 6640–12

**Figure 5:**
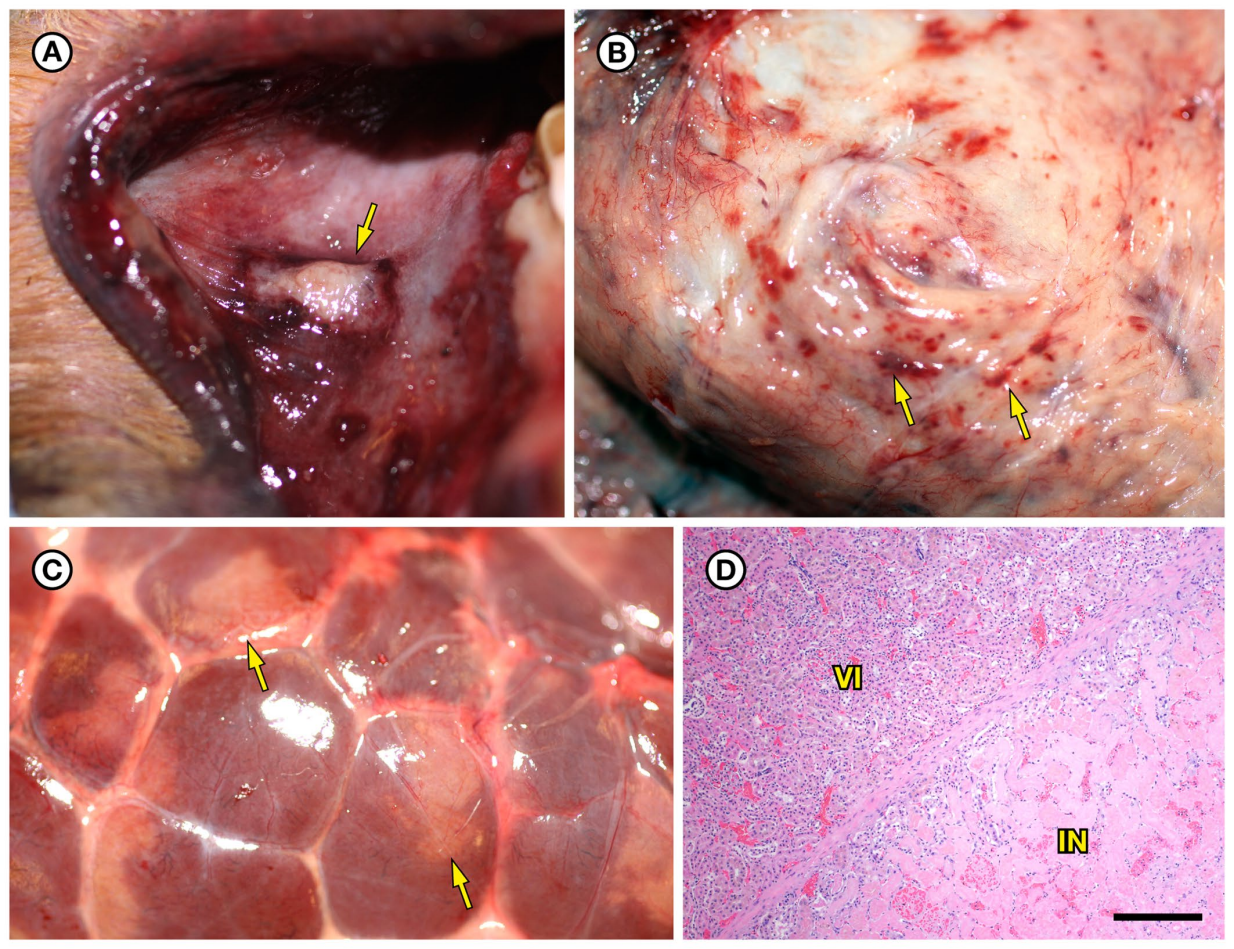
Gross and histopathologic findings from southern sea otter (*Enhydra lutris nereis*) 6640–12. **(A)** Focal rhomboidal region of mucosal infarction with hyperemic borders on the left maxillary buccal mucosa (arrow). Histopathology revealed mucosal necrosis and ulceration with a thick surface coat of mixed bacteria. The submucosa was diffusely edematous and contained small aggregates of neutrophils, macrophages, lymphocytes, and sparse hemorrhage. Perilesional submucosal blood vessels were congested, and a few contained marginated neutrophils and small intravascular fibrin thrombi. Gram stains were inconclusive for submucosal bacteria. Grossly and microscopically this lesion is compatible with the “diamond skin disease” pattern of dermal necrosis reported in pigs, cetaceans, and other animals with erysipelas. Due to the dense pelage of sea otters (the densest of all mammals), additional skin lesions could have been present, but would be easily missed at necropsy. **(B)** Inguinal subcutis from the same sea otter, showing multifocal petechia and ecchymoses (arrows). **(C)** Multifocal irregular, tan, well-demarcated renal infarcts from the same otter (arrows). *Erysipelothrix* sp. was isolated in pure culture from a renal infarct, the renal pelvis, and an axillary lymph node with no other bacteria detected. **(D)** Microscopic view of the kidney, showing viable renal parenchyma at top left (VI), compared to infarcted parenchyma at bottom right (IN). The large mass of infarcted renal parenchyma within both kidneys could have caused acute renal failure as an additional factor in this animal’s death (H&E; Bar = 220 μm).

**Figure 6:**
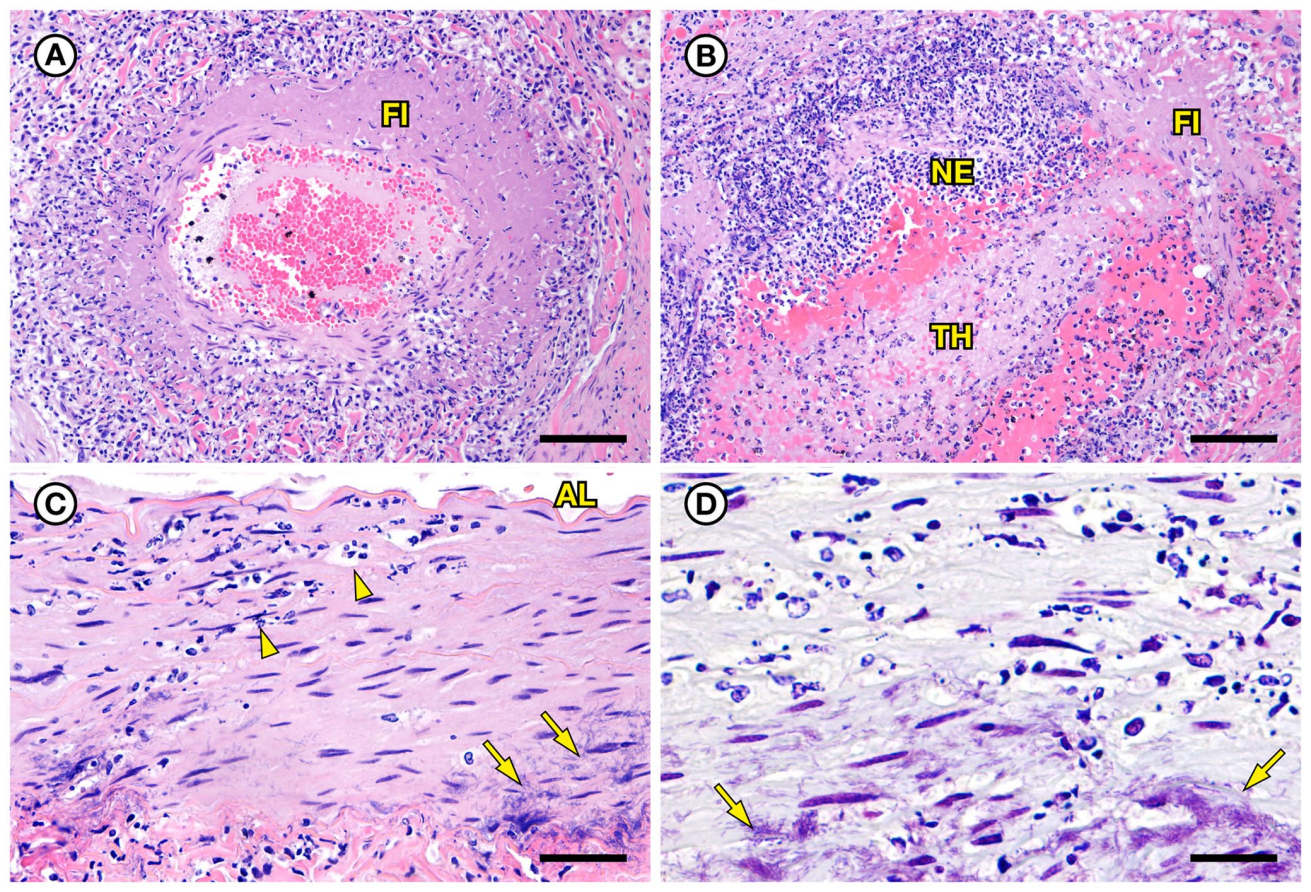
Histopathologic findings from southern sea otter (*Enhydra lutris nereis*) 6640–12. **(A)** Cross-section of renal arcuate artery from the same sea otter described in Figure 5. The arterial wall and perivascular stroma are partially obliterated by severe suppurative, histiocytic, and lymphocytic inflammation admixed with dense bands of fibrin (FI) (H&E; Bar = 92 μm). **(B)** Cross-section of a second renal arcuate artery from the same sea otter, showing a similar pattern of perivascular and vascular mural inflammation and fibrin (FI) deposition. In this profile large numbers of neutrophils (NE) are attached to the inner vascular wall, and a large fibrinosuppurative thrombus (TH) is projecting into the vessel lumen (H&E; Bar = 90 μm). **(C)** Cross-sectional view of a perirenal artery from the same otter. The arterial lumen (AL) is indicated for reference. The arterial tunica media is moderately expanded by inflammatory cells, mainly neutrophils (arrowheads). The external tunica media near the junction with the tunica adventitia contains thousands of slender bacterial rods (arrows) (H&E; Bar = 45 μm). **(D)** Gram stain of a higher magnification view of the same perirenal artery shown in **C**: Myriad intramural filamentous bacteria are spatially associated with the severe vascular mural inflammation (arrows). In this case the bacterial Gram staining is more amphophilic to red, which has been reported previously for Gram stains of *Erysipelothrix* spp. (see text of manuscript for details). *Erysipelothrix* sp. was isolated in pure culture from a renal infarct, the renal pelvis, and an axillary lymph node from this sea otter at necropsy, with no other bacteria detected (Gram; Bar = 45 μm).

SSO 6640–12 was an adult male that stranded alive and was euthanized the same day due to suspected cardiomyopathy-associated heart failure. Gross necropsy revealed a focal rhomboidal area of mucosal infarction of the left buccal mucosa (Figure 5A), severe diffuse dilation and congestion of subcutaneous blood vessels, and multifocal petechia and ecchymoses (Figure 5B). Also present were multifocal bilateral renal infarcts (Figure 5C) that were confirmed on histopathology (Figure 5D). The renal infarction was associated with severe fibrinoid necrosis and suppurative arteritis of renal and peri-renal arteries (Figure 6A), associated with vascular thrombosis and perivascular hemorrhage (Figure 6B). The walls of affected arterioles were partially effaced by intramural and perivascular fibrin, hemorrhage and suppurative inflammation, accompanied by large numbers of long, slender bacterial rods in the tunica media (Figure 6C); Gram stains revealed metachromatic to eosinophilic staining of these intramural bacilli (Figure 6D). Suppurative arteritis with intramural bacilli was also observed in a periadrenal artery and an artery near an inguinal lymph node. *Erysipelothrix* sp. were isolated in pure culture from a renal infarct, the renal pelvis, and the left axillary lymph node; no other bacterial pathogens were isolated from any tissue at necropsy. Based on findings from gross necropsy, histopathology, special stains and bacterial culture, the primary cause of death was acute bacterial sepsis due to *Erysipelothrix* sp. infection. The renal infarcts were so severe and extensive that acute renal failure may have been a sequela. Another concurrent condition that could have contributed to morbidity and mortality was mild cardiomyopathy. The animal was thin and had a chronic, healed baculum fracture with mild paraphimosis and a urine distended bladder, herpesviral glossitis and perimortem pulmonary emphysema. Potential portals for bacterial invasion in this adult male include gastric ulcers, fight trauma, and the chronic baculum fracture associated with paraphimosis and urinary retention; baculum fractures in SSOs are usually a consequence of intraspecific fight trauma (Miller et al., 2020).

#### 3.1.5 SSO 7692–15

**Figure 7:**
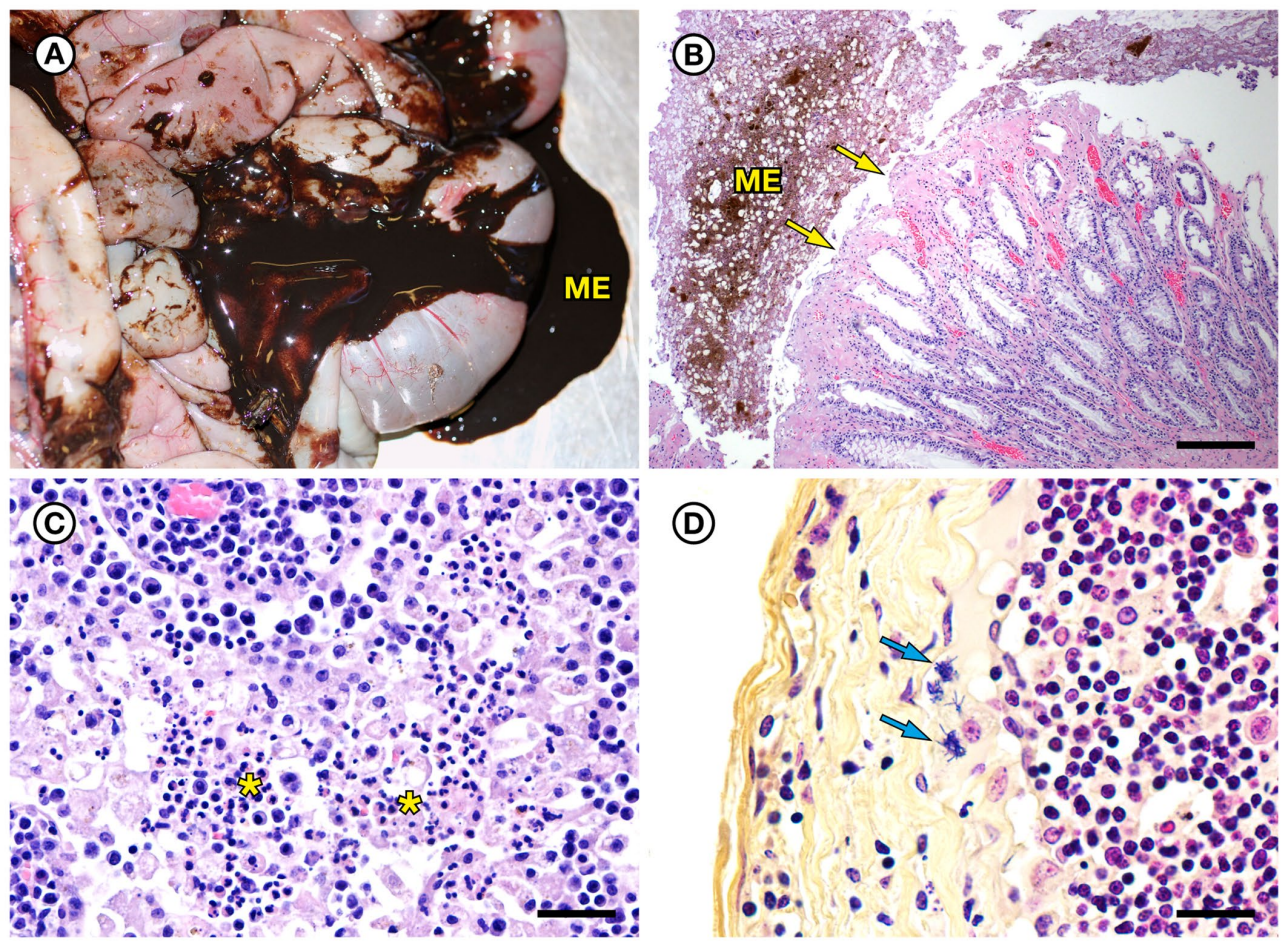
Gross and histopathologic findings from southern sea otter (*Enhydra lutris nereis*) 7692–15. **(A)** Diffuse enteritis, characterized by thickened, edematous intestinal loops and severe melena (ME). Fecal culture yielded a mixture of bacteria including *E. rhusiopathiae*. **(B)** Microscopic view of colon from the same otter: There is superficial mucosal ulceration (arrows), mild lamina proprial congestion, and marked intralumenal melena (ME) (H&E; Bar = 220 μm). **(C)** Clusters of neutrophils (asterisks) admixed with reactive macrophages in the medullary sinus of an inflamed axillary lymph node from the same otter (H&E; Bar = 40 μm). **(D)** Higher magnification Gram stain of the axillary lymph node shown in **C**. Multiple Gram-positive bacterial rods are visible within the subcapsular sinus (arrows) and are adherent to and partially phagocytized by host white blood cells (Gram; Bar = 20 μm).

SSO 7692–15 was an adult female that stranded alive, but moribund and emaciated, with a large subacute nose wound indicative of recent copulation and tarry feces; she died prior to receiving veterinary care. She had been implanted with a VHF transmitter and a TDR 5 years prior to stranding. Gross necropsy revealed diffuse gastric ulcers and moderate intestinal mural thickening and severe melena (Figure 7A). Mesenteric and peripheral lymph nodes were also enlarged, congested and meaty. Intestinal histopathology revealed mild to moderate vascular congestion, hemorrhage, and mixed suppurative, lymphoplasmacytic and mildly eosinophilic enteritis with patchy erosion and necrosis of superficial and villous epithelium (Figure 7B). However, rapid autolysis that occurs postmortem in sea otter intestine (Miller et al., 2020) precluded further assessment. This animal also had mild chronic intestinal and peritoneal *Profilicollis altmani* acanthocephalan infection; a prior study noted potential causal associations between intestinal and peritoneal acanthocephalan infections and concurrent enteritis in southern sea otters (Miller et al., 2020). The presence of a fresh nose wound, a swollen, a diffusely edematous vulva (indicative of estrus), and morphological characteristics of the nonpregnant uterus, ovaries, and mammary glands in an emaciated adult female sea otter were consistent with diagnosis of ELS as a contributing cause of death (Chinn et al., 2016). Also noted was mild bilateral pulmonary septal emphysema with a focal large bulla on the lateral aspect of the left caudal lung lobe; pulmonary emphysema is relatively common in females with significant mating-associated nasal trauma.

**Figure 8:**
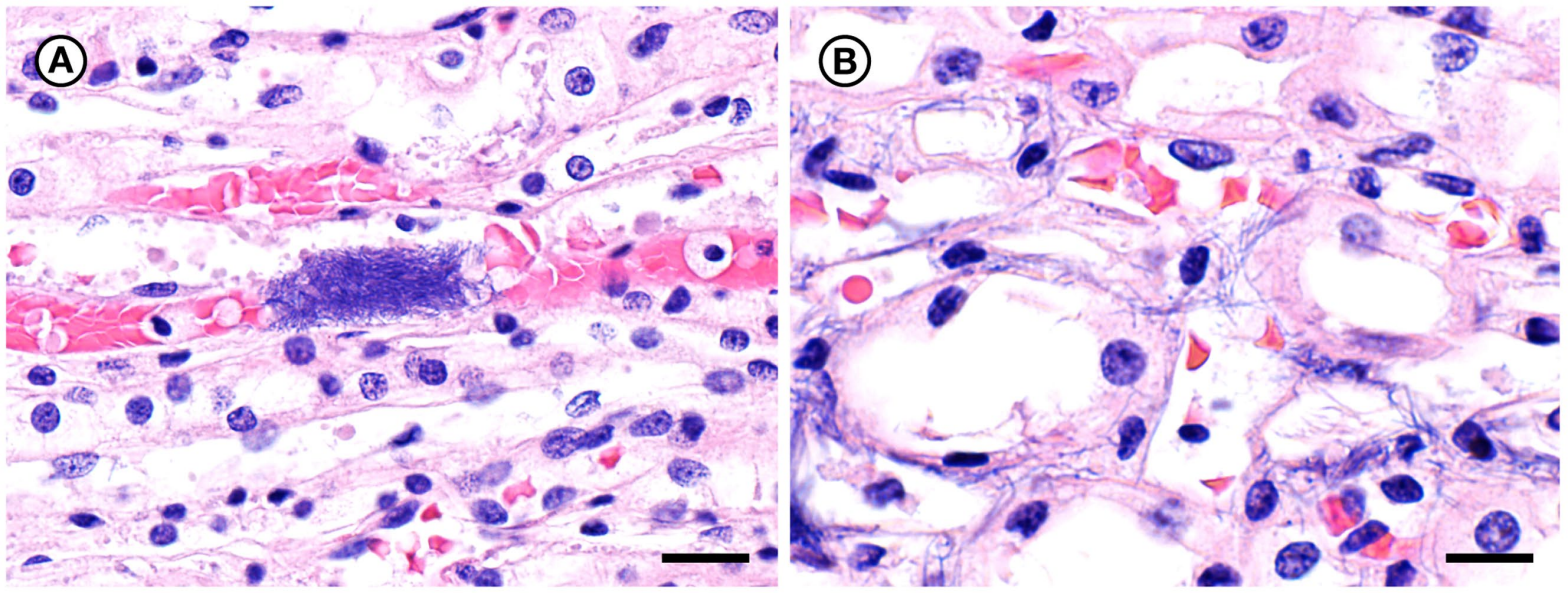
Histopathologic findings from southern sea otter (*Enhydra lutris nereis*) 7692–15. **(A)** Microscopic view of renal papilla from the same otter shown in Figure 7. A dense cluster of slender bacterial rods is visible within a capillary lumen (H&E; Bar = 20 μm). **(B)** Higher magnification view of an adjacent area of renal papilla from the same otter shown in **A**. Long, slender bacterial rods are visible within renal tubules and interstitial spaces. Gram stains of this section were negative for bacteria, suggesting that a focal area of bacterial proliferation was cut through when the block was re-surfaced in preparation for H&E recuts and Gram stains (H&E; Bar = 14 μm).

On histopathology the enlarged lymph nodes exhibited suppurative lymphadenitis (Figure 7C), and Gram stains revealed low to moderate numbers of long, slender Gram-positive bacterial rods in the subcapsular sinuses (Figure 7D). Similar bacteria were observed singly and as small clusters within capillaries of the renal papilla, as well as in the papillary interstitium and tubules (Figures 8A,B). Postmortem fecal culture yielded a mixture of *Erysipelothrix* sp., *Vibrio* spp., *V. parahaemolyticus* and *Clostridium perfringens*. No bacterial growth was obtained from the mesenteric lymph node, perhaps due to over-searing the lymph node surface as part of sterilization prior to sample collection. Based on findings from gross necropsy, histopathology, special stains and bacterial culture, the primary cause of death was mixed bacterial enterocolitis that may have been secondary to, or concurrent with mild acanthocephalan peritonitis. Prior research suggests associations between the development of bacterial enterocolitis in SSOs and concurrent *Profilicollis* spp. acanthocephalan enteritis and peritonitis (Miller et al., 2020). Based on findings from histopathology and special stains, *Erysipelothrix* sp. appears to have spread systemically as a sequela to enterocolitis. Other concurrent conditions that could have contributed to morbidity and mortality were ELS, chronic acanthocephalan enteritis and peritonitis, and anemia due to the combined effects of gastric ulcers, intestinal blood loss, and emaciation. The development of pulmonary emphysema was considered a perimortem event in an already sick animal.

### 3.2 Antimicrobial susceptibility

The five evaluated SSO *Erysipelothrix* spp. isolates had identical susceptibility profiles to the antimicrobial drugs tested except for their minimum inhibitory concentration for ceftiofur, oxytetracycline, tetracycline, sulphadimethoxine, trimethoprim/sulfamethoxazole, florfenicol, and streptomycin (Supplementary Table S4). All isolates were susceptible to penicillin and ceftiofur.

### 3.3 Surface protective antigen typing

All SSO *Erysipelothrix* spp. isolates were positive for *spaA* and negative for *spaB* and *spaC.* Likewise, amino acid sequences were homologous to spaA-type isolates from previous work (To and Nagai, 2007; Supplementary Figure S1).

### 3.4 Multi-locus sequence analysis

Three SSO isolates (6640–12, 5818–10, 3111–98) clustered with *spaA E. rhusiopathiae* isolates from birds and terrestrial mammals, including pigs, goats, and sheep, while one SSO isolate (7692–05) clustered with *spaA E. rhusiopathiae* from marine mammals and fish (Supplementary Figure S2). Two SSO isolates, 4322–04 and 4724–06 did not cluster with any of the isolates investigated.

### 3.5 Whole genome sequencing

Complete genome sequences of *Erysipelothrix* spp. strains 4322–04 and 4724–06 were deposited in GenBank (BioProject no. PRJNA776326 and PRJNA776840, respectively) under the accession nos. CP125807 and CP125808, respectively. The genome of *Erysipelothrix* sp. strain 4322–04 comprises 1,735,515 bp. The chromosome contains 1,695 proteins, 6 rRNAs, 55 tRNAs, and 4 other RNAs. Comparably, the genome of *Erysipelothrix* sp. strain 4724–06 comprises 1,782,830 bp. Identified genes on the 4724–06 chromosome encode 1,724 proteins, 7 rRNAs, 55 tRNAs, and 3 other RNAs.

The two sequenced SSO *Erysipelothrix* spp. genomes demonstrated a high degree of 16S similarity (97.5–99.9%) to *E. rhusiopathiae, E. piscisicarius*, and *E. tonsillarum* isolates; as well as an undescribed *Erysipelothrix* sp. (A18Y020d; GenBank Acc. No. NZ_OW659496) (Table 2; Zhong et al., 2022). ANI values >96% and dDDH values >70% indicate the same species (Auch et al., 2010; Meier-Kolthoff et al., 2013; Ciufo et al., 2018), while ANI values >98% and dDDH values >80% suggest the same subspecies (Meier-Kolthoff et al., 2014; Bannantine et al., 2020; Choo et al., 2020; Pearce et al., 2021). At the genome level, isolate 4322–04 shared <95% ANI to *E. rhusiopathiae* type strain ATCC 19414 (94.7%) and isolates NCTC 8163 (94.7%) and NCTC 7999 (94.6%), while exhibiting <60% dDDH similarity to the same strains (Table 3). Combined, ANI and dDDH assessments indicate isolate 4322–04 is sufficiently distant from *E. rhusiopathiae* and other *Erysipelothrix* spp. to be considered a novel taxon (Goris et al., 2007; Richter and Rosselló-Móra, 2009; Ciufo et al., 2018), most closely related (ANI: 95.6%; dDDH: 63.0%; 16S: 99.7–100%) to an undescribed *Erysipelothrix* sp. isolate (A18Y020d; GenBank NZ_OW659496) from an albatross in the Antarctic. Although some utilize >95% ANI to indicate conspecificity, multiple *Erysipelothrix* spp. show high ANI similarity but have key MLSA differences, so the more conservative ANI of 96% was elected as the species cutoff (Pomaranski et al., 2020; Grazziotin et al., 2021). The name *Erysipelothrix enhydrae* sp. nov. is proposed to represent this novel taxon.

**Table 2:** Similarity (% identity) of 16S rRNA copies of *Erysipelothrix* spp. to *Erysipelothrix enhydrae* 4322–04 and *E. rhusiopathiae* subsp. *ohloneorum* 4724–06 isolated from southern sea otters (*Enhydra lutris nereis*).

GenBank	Isolate	Submitted as:	ID^1^	# of Copies	16S Similarity (%)	
					4322–04	4724–06
*Study isolates*						
CP125807	4322–04	*E. enhydrae*	N/A	6	99.7–100	99.3–99.8
CP125808	4724–06	*E. rhusiopathiae* subsp. *ohloneorum*	N/A	7	99.3–99.8	99.4–99.9
*Erysipelothrix anatis*						
GCA_011421495	D14-1188	*E. anatis*	*E. anatis*	1	95.9–96.1	95.6–96.0
GCA_009828725	D16-1007	*E. anatis*	*E. anatis*	1	96.0–96.2	95.7–96.1
GCA_009828735	D16-1577	*E. anatis*	*E. anatis*	1	96.0–96.2	95.7–96.1
GCA_009828745	D17-0559	*E. anatis*	*E. anatis*	1	96.0–96.2	95.7–96.1
NZ_CP049860.1	HDW6B	*E.* sp.	*E. anatis*	8	96.0–96.2	95.7–96.1
*Erysipelothrix aquatica*						
NZ_WTTA00000000	181008240	*E. aquatica*	*E. aquatica*	1	96.0–96.2	95.7–96.1
NZ_WTSZ00000000	E637c	*E. aquatica*	*E. aquatica*	1	96.0–96.2	95.7–96.1
NZ_WTSY00000000	VA92-K48	*E. aquatica*	*E. aquatica*	1	96.0–96.2	95.7–96.1
*Erysipelothrix piscisicarius*						
GCA_003931795	15TAL0474	*E. piscisicarius*	*E. piscisicarius*	3	97.7–99.5	97.5–99.5
GCA_009906265	268691	*E. rhusiopathiae*	*E. piscisicarius*	1	99.7–99.9	99.5–99.9
GCA_016617625	Es2-6	*E.* sp.	*E. piscisicarius*	1	99.7–99.9	99.5–99.9
GCA_016617655	Es2-7	*E.* sp.	*E. piscisicarius*	1	99.7–99.9	99.5–99.9
*Erysiepelothrix rhusiopathiae*						
GCA_023650665	319078	*E. rhusiopathiae*	*E. rhusiopathiae*	7	99.7–99.9	99.5–99.9
GCA_022132215	10DISL	*E. rhusiopathiae*	*E. rhusiopathiae*	1	99.7–99.9	99.5–99.9
GCA_000160815	ATCC19414	*E. rhusiopathiae*	*E. rhusiopathiae*	4	99.6–99.9	99.5–99.9
GCA_000270085	Fujisawa	*E. rhusiopathiae*	*E. rhusiopathiae*	7	99.7–99.9	99.5–99.9
GCA_001602155	GXBY-1	*E. rhusiopathiae*	*E. rhusiopathiae*	9	99.4–99.9	99.3–99.9
GCA_003226675	ML101	*E. rhusiopathiae*	*E. rhusiopathiae*	5	99.6–99.9	99.5–99.9
GCA_900448055	NCTC7999	*E. rhusiopathiae*	*E. rhusiopathiae*			
GCA_900637845	NCTC8163	*E. rhusiopathiae*	*E. rhusiopathiae*	9	98.6–99.9	98.5–99.9
GCA_023221615	Poltava	*E.* sp.	*E. rhusiopathiae*	10	99.1–99.9	99.0–99.9
GCA_006384955	SE38	*E. rhusiopathiae*	*E. rhusiopathiae*	5	99.6–99.9	99.5–99.9
GCA_000404205	SY1027	*E. rhusiopathiae*	*E. rhusiopathiae*	3	99.6–99.9	99.5–99.9
GCA_003725505	VR2	*E. rhusiopathiae*	*E. rhusiopathiae*	1	99.7–99.9	99.5–99.9
GCA_001723625	WH13013	*E. rhusiopathiae*	*E. rhusiopathiae*	5	99.7–99.9	99.5–99.9
GCA_007725185	ZJ	*E. rhusiopathiae*	*E. rhusiopathiae*	6	99.6–99.9	99.5–99.9
Other *Erysipelothrix* sp.						
NZ_CP060715.1	DSM 15511	*E. inopinata*	N/A	4	95.9–96.2	95.7–96.4
NZ_CP013213.1	LV19	*E. larvae*	N/A	4	93.1–93.3	92.8–93.3
NR_113036^2^	DSM 14972	*E. tonsillarum*	N/A	1	99.4–99.7	99.3–99.7
NZ_WTTB01000018.1	165301687	*E. urinaevulpis*	N/A	1	94.8–95.0	94.7–95.1
NZ_OW659496.1	A18Y020d	*E.* sp.	*E.* sp.	7	99.7–100	99.4–99.8
NZ_CP049859.1	HDW6A	*E.* sp.	*E.* sp.	3	95.7–95.9	95.5–96.0
NZ_CP049861.1	HDW6C	*E.* sp.	*E.* sp.	5	95.2–95.4	95.0–95.5

^1^Identifications based on ANI, dDDH and phylogenetic assessment (Figure 9). ^2^No complete16S rRNA matches in *E. tonsillarum* DSM_14972 assembly GCA_000373785; alignment based on *E. tonsillarum* DSM_14972 16S rRNA nucleotide sequence NR_113036. Comparisons were based on an alignment of 1,558 bp, with the exception of *E. tonsillarum* DSM_14972, which consisted of an alignment of 1,501 bp.

**Table 3:** Average nucleotide identity and digital DNA–DNA hybridization (dDDH) comparisons for select *Erysipelothrix* spp. to *E. enhydrae* 4322–04 and *E. rhusiopathiae* subsp. *ohloneorum* 4724–06.

		ANI^1^		dDDH	
GenBank Acc. No.	Submitted as:	4322–04	4724–06	4322–04	4724–06
GCA_011421495	*Erysipelothrix anatis* D14_1188-2-1-2	<80	<80	19.6	19.6
GCA_009828725	*Erysipelothrix anatis* D16_1007-1-1-1	<80	<80	19.7	19.7
GCA_009828735	*Erysipelothrix anatis* D16_1577-8-2-2	<80	<80	19.8	20.1
GCA_009828745	*Erysipelothrix anatis* D17_0559-3-2-1	<80	<80	19.8	19.9
NZ_CP049860	*Erysipelothrix* sp. HDW6B^2^	<80	<80	21.1	21
NZ_WTTA00000000	*Erysipelothrix aquatica* 181008240	<80	<80	20	20.1
NZ_WTSZ00000000	*Erysipelothrix aquatica* E637c_KL	<80	<80	19.8	20.4
NZ_WTSY00000000	*Erysipelothrix aquatica* VA92-K48	<80	<80	22.6	22.9
GCA_003931795	*Erysipelothrix piscisicarius* 15 TAL0474	87.7	87.9	32	32.2
GCA_009906265	*Erysipelothrix rhusiopathiae* 268691^3^	87.9	87.9	31.3	31.3
GCA_016617625	*Erysipelothrix* sp. strain 2 (EsS2-6-Brazil)^3^	88.4	88.5	32.5	32.5
GCA_016617655	*Erysipelothrix* sp. strain 2 (EsS2-7-Brazil)^3^	88	88.2	31.7	31.9
GCA_022132215	*Erysipelothrix rhusiopathiae* 10DISL	94.6	97.1	56.4	76.8
GCA_023650665	*Erysipelothrix rhusiopathiae* 319078	94.6	97.3	57.6	78.4
GCA_000160815	*Erysipelothrix rhusiopathiae* ATCC 19414	94.7	97.4	57.4	78.3
GCA_000270085	*Erysipelothrix rhusiopathiae* Fujisawa	94.7	97.2	57.8	77.2
GCA_001602155	*Erysipelothrix rhusiopathiae* GXBY-1	94.6	97.3	57.9	77.2
GCA_003226675	*Erysipelothrix rhusiopathiae* ML101	94.7	97.1	57.7	77.1
GCA_900448055	*Erysipelothrix rhusiopathiae* NCTC7999	94.6	97.3	57.7	77.6
GCA_900637845	*Erysipelothrix rhusiopathiae* NCTC8163	94.7	97.3	57.7	78.6
GCA_023221615	*Erysipelothrix* sp. Poltava^4^	94.4	97.2	56.3	75.9
GCA_006384955	*Erysipelothrix rhusiopathiae* SE38	94.7	97.1	57.7	77.1
GCA_000404205	*Erysipelothrix rhusiopathiae* SY1027	94.7	97.3	57.4	77
GCA_003725505	*Erysipelothrix rhusiopathiae* VR-2	94.7	97.1	57.1	76.7
GCA_001723625	*Erysipelothrix rhusiopathiae* WH13013	94.7	97	57.7	77.1
GCA_007725185	*Erysipelothrix rhusiopathiae* ZJ	94.7	97.2	57.8	77.1
GCA_014396165	*Erysipelothrix inopinata* DSM 15511 T	<80	<80	20	20.7
NZ_CP013213	*Erysipelothrix larvae* LV19	<80	<80	22	21.5
GCA_000373785	*Erysipelothrix tonsillarum* DSM 14972	83.7	83.7	22.6	22.6
NZ_WTTB00000000	*Erysipelothrix urinaevulpis* 165301687	<80	<80	18.8	19.8
GCA_940143175	*Erysipelothrix* sp. A18Y020d	95.6	93.8	63	53.7
NZ_CP049859	*Erysipelothrix* sp. HDW6A	<80	<80	21.8	21
NZ_CP049861	*Erysipelothrix* sp. HDW6C	<80	<80	20.4	20.5

^1^FastANI reports values ~ 80–100%; No output provided for ANI values < 80%. ^2^Identified as *E. anatis* by ANI, dDDH and phylogenetic assessment (Figure 9). ^3^Identified as *E. piscisicarius* by ANI, dDDH and phylogenetic assessment (Figure 9). ^4^Identified as *E. rhusiopathiae* by ANI, dDDH and phylogenetic assessment (Figure 9). ANI values >96% and dDDH values >70% indicate same species (Auch et al., 2010; Meier-Kolthoff et al., 2013; Ciufo et al., 2018), ANI values >98% and dDDH values >80% suggest same subspecies (Meier-Kolthoff et al., 2014; Bannantine et al., 2020; Choo et al., 2020; Pearce et al., 2021).

**Figure 9:**
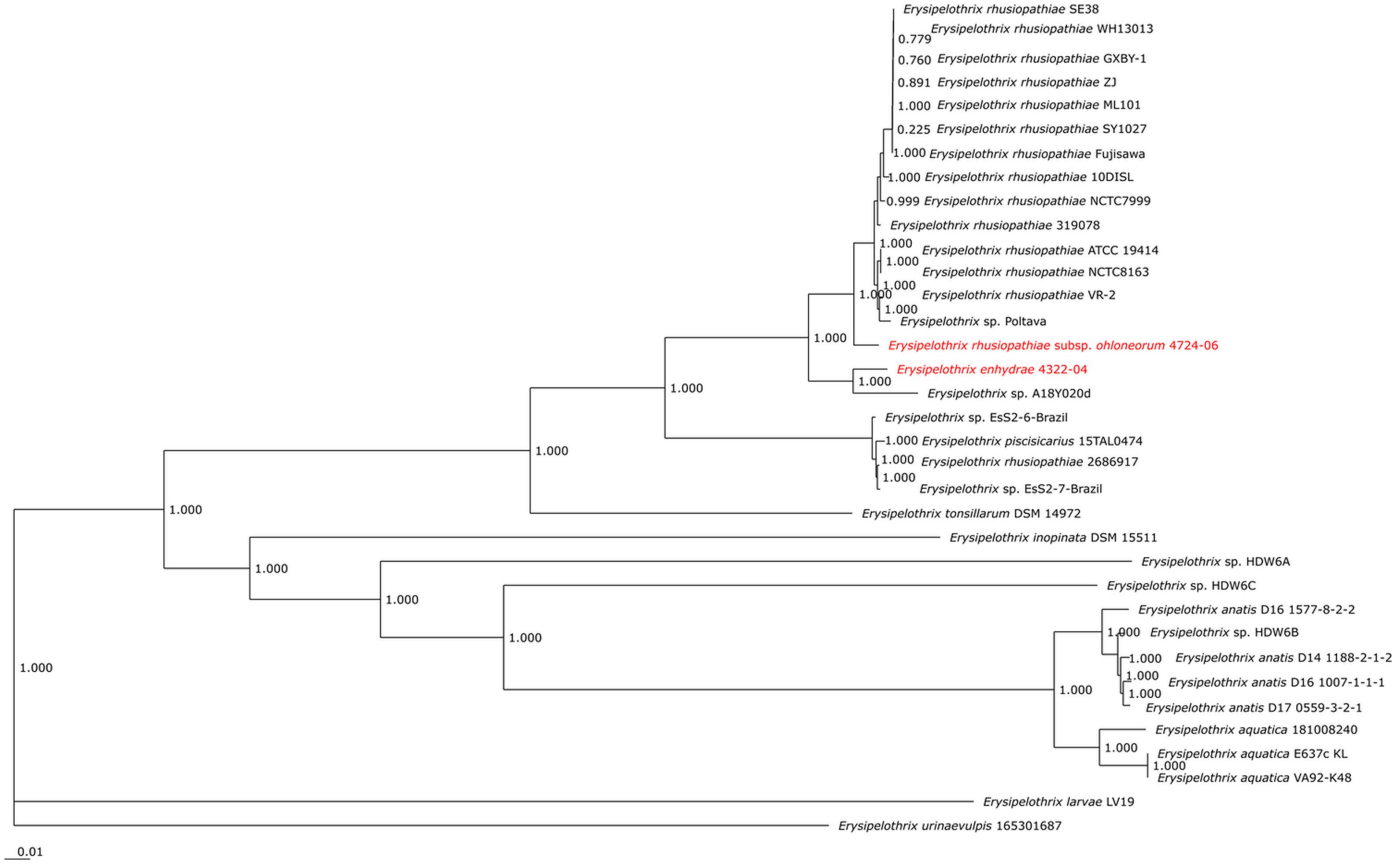
Neighbor-joining phylogeny built from 35 *Erysipelothrix* spp. core genomes consisting of 428 genes per genome. *Erysipelothrix enhydrae* 4322–04 and *E. rhusiopathiae* subsp. *ohloneorum* 4724–06 from southern sea otters (*Enhydra lutris nereis*) characterized in the current study are highlighted in red text. GenBank Accession numbers for genome assemblies used in this figure are provided in Table 3.

The second isolate, 4724–06, shared 95.3% ANI and 59.1% GGD to isolate 4322–04 but increased similarity to *E. rhusiopathiae* type strains ATCC 19414 (ANI: 97.4%; dDDH: 78.3%), NCTC 8163 (ANI: 97.3%; dDDH: 78.6%) and NCTC 7999 (ANI: 97.3%; dDDH: 77.6%), with ANI (>95%; <98%) and dDDH (>70%; <80%) values in the subspecies range for the three *E. rhusiopathiae* type strains (Bannantine et al., 2020; Choo et al., 2020; Pearce et al., 2021), suggesting isolate 4724-06 represents a novel subspecies. Herein, the name *Erysipelothrix rhusiopathiae ohloneorum* ssp. nov is proposed for isolate 4724–06. Both ANI and dDDH affiliations were supported by neighbor-joining phylogenies built from the complete core genome (403,764 bp; 428 genes) (Figure 9).

### 3.6 Fatty acid and biochemical characterization

Biochemical characterization of eight *Erysipelothrix* isolates, including the two novel *Erysipelothrix* spp. isolates from SSOs, demonstrated that the isolates share a homogeneous profile (Supplementary Table S5). The exception being isolate 4322–04, which produced three different reactions: negative for Beta glucuronidase (βGUR), weak positive for Voges Proskauer (VP), and positive for Tagatose (TAG).

FAME analysis of the *Erysipelothrix* spp. isolates demonstrated each shared a core group of fatty acids (Supplementary Table S6). These fatty acids were 16:0, 18:1 ω9c, 18:0, 20:4 ω6,9,12,15c, Summed Feature 3, Summed Feature 5, and Summed Feature 8 (Supplementary Table S6). *Erysipelothrix* sp. 4322–04 was the only isolate to produce the fatty acids 17:1 ω8c and 20:0 iso and *Erysipelothrix* sp. 4724–06 was the only isolate to produce the fatty acid 17:1 iso ω5c. *E. rhusiopathiae* 7155 and 7692–15 were the only isolates to produce the fatty acid, 8:0 3OH. *E. rhusiopathiae* isolate H1T1 and *E. piscisicarius* 15TAL064 did not produce the fatty acid 14:0.

### 3.7 Tiger barb *in vivo* challenge

**Figure 10:**
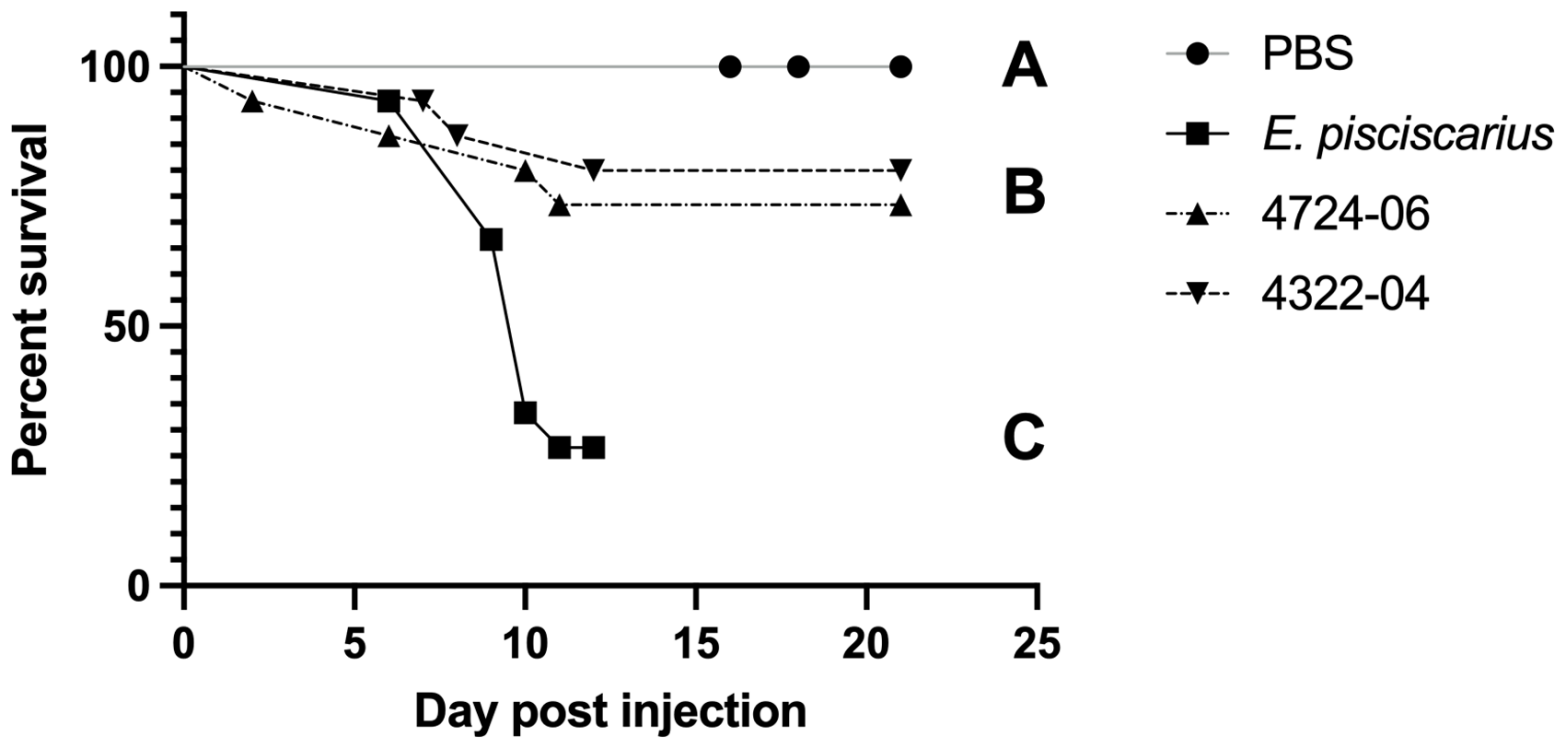
Kaplan–Meier survival curve for tiger barbs (*Puntigrus tetrazona*) challenged with novel *Erysipelothrix* spp. isolates (4322–04 *Erysipelothrix enhydrae* sp. nov. and 4724–06 *E. rhusiopathiae ohloneorum* ssp. nov.) from necropsied southern sea otters (*Enhydra lutris nereis*). Controls include phosphate-buffered saline (PBS) as a negative control and a well-characterized strain of *Erysipelothrix piscisicarius* that causes mortality in tiger barbs (Pomaranski et al., 2020). Different letters connote a significant difference between treatments. One fish in 4724–06 was unaccounted for, possibly consumed by tankmates.

Mortality in fish (TBs) exposed to the unique *E. rhusiopathiae ohloneorum* ssp. nov. isolate 4724–06 and *E. enhydrae* sp. nov. isolate 4322–04 began on day two and seven and continued through day 11 and 12, respectively (Figure 10). There were four recorded mortalities (4/15) in TB exposed to *E. rhusiopathiae ohloneorum* ssp. nov. isolate 4724–06, three mortalities (3/15) in TB exposed to *E. enhydrae* sp. nov. isolate 4322–04, and 11 mortalities (11/15) in TBs exposed to *E. piscisicarius* over the course of the experiment. At the conclusion of the 21-day trial, TBs exposed to *E. rhusiopathiae ohloneorum* ssp. nov isolate 4724–06 had a 73.3% probability of survival, compared to 80% for TBs exposed to *E. enhydrae* sp. nov. isolate 4322–04.

Bacteria were successfully re-isolated from the brains following death for 8/12 TBs challenged with 4322–04 *E. enhydrae* sp. nov.; 4/12 of the isolates were confirmed to be *Erysipelothrix* spp. via PCR. There was no successful re-isolation of *Erysipelothrix* spp. from fish challenged with SSO 4724–06 *E. rhusiopathiae ohloneorum* ssp. nov. Colonies morphologically consistent with *Erysipelothrix* spp. were isolated from a fish that died following challenge with the SSO 4724–06 *E. rhusiopathiae ohloneorum* ssp. nov. strain, but these bacterial colonies were not *Erysipelothrix* sp.-positive via 16S rRNA PCR.

**Figure 11:**
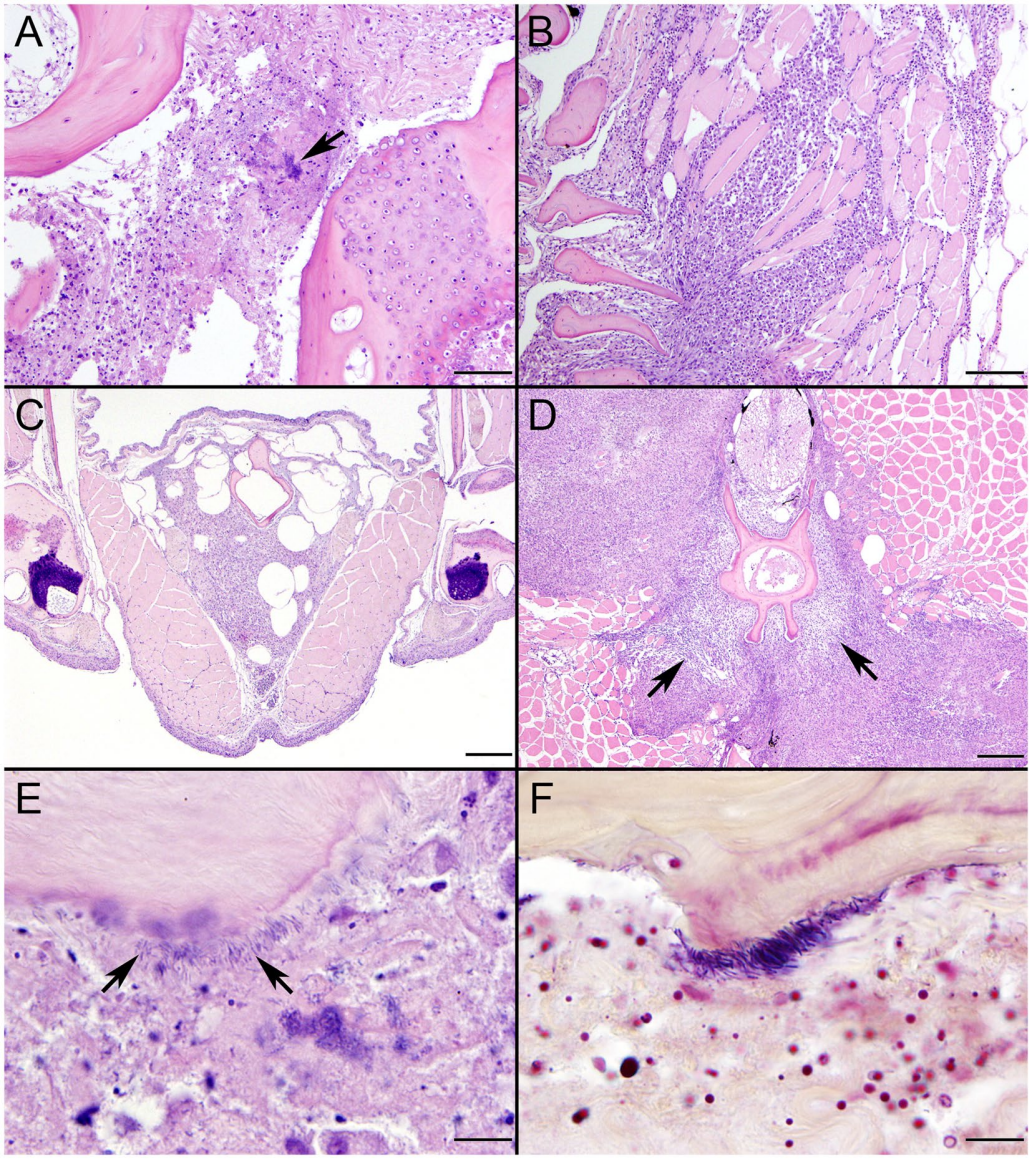
Histopathologic changes in tiger barbs (*Puntigrus tetrazona*) challenged by intracoelomic injection with novel southern sea otter (*Enhydra lutris nereis*) *E. rhusiopathiae ohloneorum* ssp. nov. isolate 4724–06. **(A)** Abundant cellular debris, consistent with acute necrosis, replaces collagen fibers between two bones at the base of a pectoral fin 11 days post challenge. The arrow indicates a microcolony of bacteria rods (H&E; Bar = 50 μm). **(B)** Intense granulomatous inflammatory infiltrates isolate and replace skeletal myofibers adjacent to bone of a pectoral fin 10 days post challenge (H&E; Bar = 100 μm). **(C)** Sheets of macrophages laden with cellular debris persist within the isthmus area 21 days after bacterial challenge surrounding bone and replacing adipose tissue (H&E; Bar = 200 μm). **(D)** Proliferating fibroblasts (arrows) envelop vertebral bone and are bordered by fields of granulomatous inflammation that replaces extensive regions of skeletal muscle, infiltrates the spinal canal, and partially surrounds the spinal cord. Small numbers of scattered bacterial rods and cellular debris remained within the inflammatory cell infiltrate after 21 days (H&E; Bar = 200 μm). **(E)** Necrotic debris and bacterial rods (arrows) border the scalloped margin of lytic bone in a dorsal fin 11 days after challenge (H&E; Bar = 10 μm). **(F)** A Gram stain highlights Gram-positive bacterial rods, consistent with *Erysipelothrix* sp., lining bone (Brown & Hopps; Bar = 10 μm).

**Figure 12:**
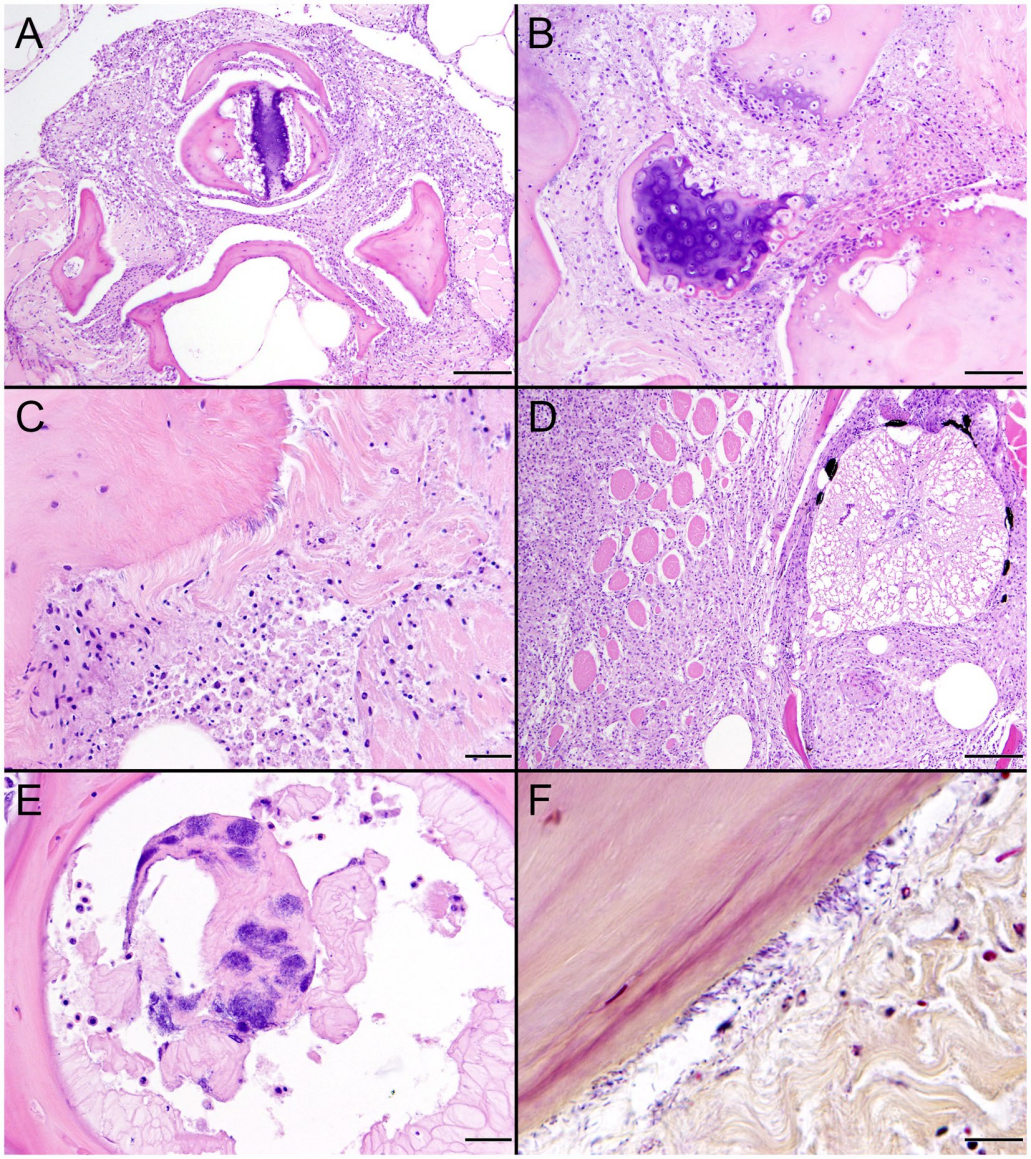
Histopathologic changes in tiger barbs (*Puntigrus tetrazona*) challenged by intracoelomic injection with novel southern sea otter (*Enhydra lutris nereis*) *Erysipelothrix enhydrae* sp. nov. isolate 4322–04. **(A)** Granulomatous inflammation and necrotic cellular debris envelop bone of pterygiophores at the base of a dorsal fin 7 days post challenge (H&E; Bar = 100 μm). **(B)** Necrotic debris and collagenolysis surrounds bones at the base of a pectoral fin. Soft tissue changes are accompanied by chondrolysis and osteolysis of two articular surfaces with irregular scalloped margins 8 days post challenge (H&E; Bar = 50 μm). **(C)** Necrotic cellular debris and collagenolysis adjacent to bone at a dorsal fin base. Small numbers of inflammatory cells infiltrate remaining collagen. A portion of the bone is lined by bacterial rods lying parallel to collagen fibers 8 days after challenge (H&E; Bar = 20 μm). **(D)** Intense granulomatous inflammation infiltrates the spinal canal, surrounds the spinal cord, and isolates and replaces skeletal myofibers within epaxial muscle 21 days post challenge (H&E; Bar = 100 μm). **(E)** Remnants of intervertebral notochord containing large numbers of bacterial rods (H&E; Bar = 20 μm). **(F)** A Gram stain illustrates Gram-positive bacterial rods, consistent with *Erysipelothrix* spp., lining bone and scattered in adjacent collagen (H&E; Bar = 10 μm).

Histopathologic changes induced by the two novel *Erysipelothrix* spp. in TBs were notably similar. Infections localized predominantly at fin bases and in the isthmus region with particular affinity for fibrous connective tissue and bone surfaces (Figures 11A–C, 12A–C). Lesions were initially lytic and necrotizing resulting in collagenolysis, chondrolysis, and osteolysis often in the presence of abundant cellular debris derived primarily from infiltrating inflammatory cells (Figures 11A,B, 12B,C). Inflammatory cell infiltrates were dominated by neutrophils on days 6–8 (Figures 11B, 12A–C), by macrophages on days 10–12, and mixtures of macrophages and lymphocytes on day 21 (Figures 11C,D, 12D). Inflammatory changes were often accompanied by fibroplasia surrounding bone in fish surviving to day 21 (Figure 11D). Short to elongate bacterial rods were variably present in microcolonies, scattered among necrotic debris and inflammatory infiltrates, and along the surfaces of bone often lying parallel to collagen fibers (Figures 11A,E, 12E). In the two challenge groups, bacteria persisted in fish surviving until day 21, both in close association with bone and scattered in areas of granulomatous inflammation (Figures 11D, 12E). In one fish from each group, inflammatory changes entered the spinal canal and partially enveloped the spinal cord (Figures 11D, 12D). Involvement of skeletal muscle was uncommon and when present appeared as an extension of primary bone and periarticular lesions (Figures 11A,D, 12D). The bacteria stained strongly Gram-positive and were best visualized using the B&H stain (Figures 11F, 12F).

## 4 Discussion

### 4.1 Gross necropsy, histopathology, special stains, and bacterial culture

The objectives of the current study were to estimate the frequency of detection of *Erysipelothrix* spp. in dead SSOs, describe pathology associated with *Erysipelothrix* spp. infection, characterize the genetic diversity and antimicrobial sensitivity of SSO *Erysipelothrix* spp. isolates, and evaluate the virulence of novel *Erysipelothrix* spp. using an *in vivo* fish model. Low incidence of *Erysipelothrix* spp. infections in SSOs may be due to proliferation of other bacteria that outcompete *Erysipelothrix* spp. growth *in vitro* and sampling bias, as only grossly apparent lesions were submitted for bacterial culture. However, fecal, oral, and rectal swabs from live, ostensibly healthy SSOs sampled during routine capture and tagging activities revealed no isolates from live SSOs, suggesting that *Erysipelothrix* spp. are uncommon as commensal flora (Dudek et al., 2022; N. Dudek, pers. comm.).

Based on our samples, both *Erysipelothrix* spp. and *E. rhusiopathiae* appeared to be opportunistic SSO pathogens. Although no clear differences were identified with respect to the infecting *Erysipelothrix* species or genotype and observed SSO lesion patterns, our current sample size is too small for definitive conclusions; assessment should be repeated when larger sample sizes are available.

*Erysipelothrix* sp. infection was considered a primary or contributing cause of death for all six SSOs, and *Erysipelothrix* infection, alone or in combination with other bacteria was a primary cause of death for at least four cases (Table 1). *Erysipelothrix* spp. were isolated in pure culture in three cases and septicemia was diagnosed as a primary or contributing cause of death in five of the six infected SSOs (Table 1).

When the five SSOs with detailed case information were compared, some interesting lesion patterns emerged. Enlarged, reactive lymph nodes and/or suppurative lymphadenitis were reported in all cases. *Erysipelothrix* spp. were isolated from one or more of the enlarged lymph nodes during necropsy from four of the five SSOs, and long, slender Gram-positive bacteria were observed within affected lymph nodes in two cases.

Fibrinosuppurative arteritis and periarteritis, with perivascular hemorrhage and intramural filamentous bacterial rods were observed microscopically in two of the five *Erysipelothrix* spp.-infected SSOs, especially in renal and perirenal arteries (Figures 2, 6). These lesions were accompanied by vascular thrombosis and renal (Figures 5C,D) and splenic infarcts. Renal pathology is also common in teleost fish infected with *E. piscisicarius* (Pomaranski et al., 2018). Long, slender Gram-positive or Gram-variable bacterial rods were observed in the tunica intima of inflamed arteries in both cases, and *Erysipelothrix* spp. were isolated at necropsy, either alone or in combination with other bacteria. Bacterial arteritis has been previously reported in *Erysipelothrix* spp.-infected animals, and this organism has been proposed for use in rodent models of arteritis (Nakato et al., 1985; Shinomiya and Nakato, 1985). *Erysipelothrix* spp.-associated mortality with arteritis was also reported during a recent epizootic in muskoxen (*Ovibos moschatus wardi*) in the Canadian Arctic; the unusual occurrence of this event in an arctic species was attributed to climate change (Kutz et al., 2015). *Erysipelothrix rhusiopathiae*-associated vasculitis and thrombosis has also been reported in southern right whale calves (*Eubalaena australis*) in Argentina (Fiorito et al., 2016). The putative source of infection in the whales was gull-inflicted skin wounds. Skin trauma is a common route for *Erysipelothrix* spp. infection in humans and other animals (Connell et al., 1952; Reboli and Farrar, 1989; Veraldi et al., 2009). Although examined SSOs in the current study had evidence of external trauma, no single route of bacterial invasion could be identified due to the concurrent presence of other potential routes of bacterial invasion, including gastric ulcers, dental disease, enteritis and/or acanthocephalan peritonitis. A larger sample size would be needed to address this question as part of future studies.

Another interesting lesion presentation in the current study was bilateral suppurative uveitis, scleritis, and endophthalmitis with hypopyon, likely resulting in blindness (Figure 3); *Erysipelothrix* sp. was isolated in pure culture from anterior chamber fluid from the affected SSO. Bilateral endophthalmitis and blindness secondary to *E. rhusiopathiae* infection was also reported in a 62-year-old man in England; bacterial exposure was associated with handling raw pork (Elvy et al., 2008). *Erysipelothrix rhusiopathiae* was also isolated from a horse with uveitis, acute blindness, laminitis, localized ventral edema and depression (Seahorn et al., 1989). The horse failed to respond to therapy and died 96 h after the onset of clinical signs. Collectively these cases may suggest tropism by *Erysipelothrix* spp. for the eyes of infected people and animals.

One *Erysipelothrix* sp.-infected SSO had a focal discrete, rhomboidal expanse of mucosal infarction with congested borders on the left maxillary buccal mucosa (Figure 5A). Although not definitive, this lesion was grossly and microscopically compatible with the “diamond skin disease” pattern of dermal necrosis described from pigs, cetaceans, dogs, birds, and other animals with erysipelas (Grieco and Sheldon, 1970; Melero et al., 2011; Marshall et al., 2019; Habte et al., 2021; Malik et al., 2021). Due to the extremely dense pelage of sea otters (this species has the densest fur of all mammals; (Williams et al., 1992)), additional rhomboidal lesions could have been present on the skin of one or more *Erysipelothrix*-infected SSOs in the current study but would be easily missed at necropsy. However, the SSO with focal mucosal infarction also had extensive subcuticular petechia and ecchymoses (Figure 5B).

Also noted was a case of severe bacterial meningoencephalitis and ventriculitis (Figure 1); mixed bacteria, including *Erysipelothrix* sp., were isolated from multiple sites, and several types of bacteria, most commonly long, slender Gram-positive bacilli, were present within the brain lesions on histopathology. Although the gross lesions in SSO 5818–10 were less obvious, when compared with other SSO cases, *Erysipelothrix* sp. was isolated in pure culture from multiple lymph nodes and diffuse suppurative lymphadenitis was observed on histopathology (Figure 4B). Peracute sepsis was considered the primary cause of death for that case; observed diffuse brain swelling and multifocal herniation (Figure 4A) was attributed to severe brain congestion and perivascular to diffuse edema, likely secondary to a sepsis-associated vasculopathy. Interestingly, pigs that die from acute erysipelas commonly present as sudden deaths without significant gross lesions (Habte et al., 2021); this case may represent a similar clinical pattern.

Other common, but relatively non-specific findings in *Erysipelothrix* spp.-infected SSOs included severe, diffuse dilation and congestion of submucosal blood vessels and pulmonary septal emphysema; each condition was present in three of the five cases. Diffuse subcutaneous vasodilation is a nonspecific expression of many different disease processes, including septicemia, endotoxemia, hyperthermia, and shock. Multifocal to diffuse congestion and multifocal hemorrhage has been reported from other animals with fatal erysipelas, including stranded cetaceans (Ceccolini et al., 2021). Similarly, septal emphysema is common in lungs of sea otters dying from diverse causes, including petroleum exposure, mating-associated trauma to the nose and face, coccidioidomycosis, and abnormal respiration due to central nervous system pathology (Miller et al., 2020); neither condition is specific to *Erysipelothrix* spp. infection in SSOs. Although vegetative valvular endocarditis has been described in association with *Erysipelothrix* spp. infection in humans and other animals (Gorby and Peacock, 1988; Cabrera-García et al., 2020), it was not observed in infected SSOs, possibly due to small sample size rather than differing pathogenesis.

Variable (Gram-positive, Gram-negative, or metachromatic) Gram staining for *Erysipelothrix* spp., as noted for SSO 6640–12 in the current study (Figure 6D), has been reported elsewhere (Dunbar and Clarridge, 2000; McLauchlin and Riegel, 2012; Jean et al., 2019). In the current study, cumulative results from bacterial culture and histopathology strongly support the identity of the Gram-variable, long, slender vascular mural bacterial rods as *Erysipelothrix* sp.; this bacterium was isolated in pure culture from multiple sites and the bacterial morphology and observed lesion patterns were comparable between SSO *Erysipelothrix* spp. cases. *Erysipelothrix* spp. bacterial morphology is also noted in different manuscripts as short bacilli or long, slender, filamentous rods (Reboli and Farrar, 1989; Dunbar and Clarridge, 2000; Prod’Hom and Bille, 2010; Jean et al., 2019). In the current study they usually appeared as long, slender bacilli (6640–12: Figures 6C,D and 7692–15: Figure 8), but appeared shorter in some Gram stains (4322–04: Figure 1E). Some *Erysipelothrix* spp. may appear more filamentous than others (Reboli and Farrar, 1989) and long filamentous forms resembling lactobacilli have been reported (Dunbar and Clarridge, 2000; McLauchlin and Riegel, 2012). Finally, *Erysipelothrix* spp. can form long chains during cell reproduction (Reboli and Farrar, 1989; Dunbar and Clarridge, 2000; McLauchlin and Riegel, 2012), contributing to their filamentous appearance. The variable appearance of these bacteria on histopathology, direct smears, and Gram stains is important to keep in mind during review of potential cases of *Erysipelothrix* spp.-associated morbidity and mortality.

### 4.2 Antimicrobial susceptibility

The five evaluated SSO *Erysipelothrix* spp. isolates showed similar susceptibility profiles to each other, including susceptibility to two of the recommended antibiotics for *E. rhusiopathiae* infections, penicillin and ceftiofur (Wang et al., 2010; Opriessnig et al., 2013; CLSI, 2017). When compared to *spaC* isolates from ornamental fish, two of the SSO *Erysipelothrix* spp. isolates had higher MICs for florfenicol, but otherwise had similar or lower MICs. The tested SSO isolates had similar MICs to isolates from various terrestrial and aquatic animals and humans (Venditti et al., 1990; Fidalgo et al., 2002; Eriksson et al., 2009). Based on these results, penicillin appears as an appropriate first line antibiotic for treating rehabilitated SSOs with *Erysipelothrix* sp. infections, but should be confirmed with culture and susceptibility profiles.

### 4.3 Surface protective antigen typing

To genetically characterize *Erysipelothrix* spp. isolates, we first identified their *spa* type. This surface protein has high immunogenic properties and *spa*-focused immunization can provide host protection against *Erysipelothrix* infections; thus, it is one of the most promising vaccine candidates (Janßen et al., 2015). Previous studies found that marine mammal *E. rhusiopathiae* isolates exhibit more heterogeneity in surface protective antigen (expressing both *spaA* and *spaB* isoforms), whereas terrestrial animals only possessed the *spaA* isoform (Opriessnig et al., 2013; Janßen et al., 2015; Pomaranski et al., 2018). Fish *E. rhusiopathiae* isolates possess both the *spaA* and *spaB* isoforms, though isolates from *E. piscisicarius* systemically infected fish are usually *spaC*-positive (Pomaranski et al., 2020). Although *spaA*-derived vaccines might help prevent *Erysipelothrix* spp. infections in SSOs because they all possess the *spaA* isoform, this apparent homogeneity could also be an artifact of limited sample size.

### 4.4 Multi-locus sequence analysis and whole genome sequencing

Although all six SSO isolates had the *spaA* genotype, only four clustered with known *spaA-*positive *E. rhusiopathiae* isolates from terrestrial animals, marine mammals, and fish without systemic infections (Supplementary Figure S2). The sequence of genes examined for three SSO isolates were most closely related to *Erysipelothrix* spp. strains derived from terrestrial animals, while the fourth was more closely associated with isolates from marine mammals and fish. Although there were clusters of isolates from the terrestrial and marine environments, this may be due to sample inclusion bias, as other work has shown a wide range of host species within *E. rhusiopathiae* clades (Forde et al., 2016). *SpaB* isolates tend to be isolated from marine sources, but further work is needed to elucidate any significance of the relatedness of the SSO isolates to strains derived from terrestrial versus marine animals (Forde et al., 2016).

The remaining two isolates (from SSOs 4322–04 and 4724–06) did not cluster with any other isolates and were identified as a new species and new subspecies, respectively, via whole genome sequencing. Biochemically, the novel species *E. enhydrae* was distinct from all other isolates, while the novel subspecies *E. rhusiopathiae ohloneorum* was identical biochemically to known *E. rhusiopathiae* isolates. Biochemical comparison on the other *spaA* isolates with whole genome sequences available (Fujisawa, GXBY-1, and SY1027) was not performed.

### 4.5 Tiger barb *in vivo* challenge

To further characterize the two novel SSO *Erysipelothrix* spp. isolates, *in vivo* challenge was performed using a previously validated TB model (Pomaranski et al., 2020). Experimental infection studies have shown that *E. rhusiopathiae* strains are infectious to multiple host species; marine environment and fish isolates can induce cutaneous lesions in pigs (Opriessnig et al., 2013). Different clades of *E. rhusiopathiae* contain isolates from taxonomically varied host species and it is probable that all lineages can infect a wide range of species (Forde et al., 2016). Thus, the TB model is useful to study isolates recovered from other host species, but does not replace future work to assess virulence of the SSO isolates, such as *in vitro* challenge using a mammalian cell line or an *in vivo* rodent model.

Post-infection mortalities occurred in TBs exposed to both novel SSO isolates, although infection was only confirmed via bacterial isolation and PCR from fish infected with *E. enhydrae* sp. nov. isolate 4322–04. Bacterial isolates that grew but were not confirmed to be *Erysipelothrix* spp. may have been opportunistic bacteria as part of a mixed bacterial infection that outgrew the slower growing *Erysipelothrix* spp. Lower morbidity and mortality were observed in TBs injected with either SSO *Erysipelothrix* sp. isolate than those challenged with *E. piscisicarius* via intracoelomic injection, or *spaA E. rhusiopathiae* by immersion (Pomaranski et al., 2020). However, these *Erysipelothrix* spp. isolates that caused higher fish mortality were originally obtained from fish, and *E. piscisicarius* was isolated from clinically ill fish (Pomaranski et al., 2018, 2020). Although pathogenesis of *Erysipelothrix piscisicarius* infections have not been clearly elucidated in mammals or birds, infections by genetically similar species, as determined by dDDH and ANI, have been reported in swine and turkeys and *E. piscisicarius* was therefore elected as a positive control (Grazziotin et al., 2021).

Although the SSO isolates did not cause as high mortality as *E. piscisicarius*, they did induce some similar lesions to *E. piscisicarius*; both bacterial isolates had an affinity for connective tissues, primarily the surfaces of bone and associated periarticular collagen of the fin bases, isthmus, and vertebral column (Pomaranski et al., 2020). Unlike *E. piscisicarius*, neither bacterium entered the coelomic cavity, affected parenchymal organs, or migrated along mesenteric and perivascular connective tissues. Severe ulcerative dermatitis was not observed and changes in the skin were limited to small numbers of neutrophils or lymphocytes scattered in dermal connective tissues or scale pockets, often adjacent to more significant lesions associated with fins and isthmus. The widespread presence of bacterial microcolonies in the dermis and interstitial areas of skeletal muscle typical of *E. piscisicarius* infection was not seen. Bacteria stained strongly Gram-positive and like *E. piscisicarius* were pleomorphic in tissue sections, varying from short to filamentous, similar to the findings from infected SSOs. In H&E-stained sections, small numbers of filamentous forms lying parallel to collagen fibers could easily be overlooked.

Although *E, rhusiopathiae* is found on the external surface of fish, it does not typically cause systemic disease in teleosts (Opriessnig et al., 2013), whereas *E. piscisicarius* infection can cause systemic disease and death (Pomaranski et al., 2018, 2020). *Erysipelothrix enhydrae* sp. nov. and *E. rhusiopathiae ohloneorum* ssp. nov. may have the potential to cause primary or opportunistic infections in fish, humans, and other animals; further investigation is merited. Future studies should investigate the effectiveness of immersion challenge in fish, which more closely represents the natural route of infection, the effect of varying environmental conditions (e.g., temperature and salinity), the role of skin wounds in pathogenesis, and whether these opportunistic pathogens can easily spread between infected fish and animals.

### 4.6 Conclusion

Our findings include the first description of pathology associated with *Erysipelothrix* spp. infection in SSOs and the identification of a novel *Erysipelothrix* species and *E. rhusiopathiae* subspecies. Although uncommonly isolated from SSOs, *Erysipelothrix* spp., alone or in combination with other bacteria were a primary or contributing cause of death for all SSOs in this study. Pathologic lesions associated with *Erysipelothrix* spp. in the SSOs varied, but encompassed lesions typically associated with *E. rhusiopathiae* infections in other species, including rhomboidal mucosal infarction (similar to “diamond skin disease”), arteritis and vasculitis, and lymphadenitis. Other lesions seen in the SSOs are only occasionally reported in other species, such as ocular pathology, meningoencephalitis, and ventriculitis. A novel *Erysipelothrix* species, *E. enhydrae* sp. nov., and subspecies, *E. rhusiopathiae ohloneorum* ssp. nov., were isolated and characterized from SSOs and both caused infection and mortality in a TB fish model, indicating the virulence of this isolate *in vivo*. All isolates showed similar antimicrobial susceptibility to isolates from other terrestrial and aquatic species, including susceptibility to recommended first-line antibiotics. As more *Erysipelothrix* spp. isolates are recovered and characterized from SSOs, we will be able to draw more conclusions about the diversity of *Erysipelothrix* spp. infections in marine mammals and the threat they may pose to the threatened SSOs and humans.

## Data availability statement

The datasets presented in this study can be found in online repositories. The names of the repository/repositories and accession number(s) can be found in the article/Supplementary material.

## Ethics statement

The animal study was approved by University of California-Davis Institutional Animal Use and Care Committee. The study was conducted in accordance with the local legislation and institutional requirements.

## Author contributions

RC: Formal analysis, Investigation, Methodology, Writing – original draft, Writing – review & editing. MAM: Conceptualization, Data curation, Formal analysis, Funding acquisition, Investigation, Methodology, Project administration, Resources, Supervision, Validation, Visualization, Writing – original draft, Writing – review & editing. HT: Data curation, Formal analysis, Investigation, Investigation, Writing – review & editing. DR: Formal analysis, Investigation, Writing – review & editing. JG: Data curation, Investigation, Methodology, Writing – review & editing. BL: Data curation, Formal analysis, Investigation, Methodology, Writing – review & editing. CO: Data curation, Investigation, Methodology, Writing – review & editing. GW: Data curation, Formal analysis, Investigation, Methodology, Writing – review & editing. EP: Data curation, Formal analysis, Investigation, Methodology, Visualization, Writing – review & editing. KS: Formal analysis, Investigation, Methodology, Writing – review & editing. AC: Conceptualization, Data curation, Formal analysis, Investigation, Methodology, Resources, Visualization, Writing – review & editing. FB: Formal analysis, Investigation, Methodology, Writing – review & editing. BB: Data curation, Investigation, Methodology, Resources, Writing – review & editing. MJM: Investigation, Resources, Writing – review & editing. MG: Data curation, Investigation, Methodology, Resources, Supervision, Writing – review & editing. ES: Conceptualization, Data curation, Formal analysis, Funding acquisition, Investigation, Methodology, Project administration, Resources, Supervision, Visualization, Writing – review & editing.

## Footnotes

^1^https://github.com/ParBLiSS/FastANI/releases

^2^https://ggdc.dsmz.de/distcalc2.php

## Appendix A

### Species descriptions

*Erysipelothrix enhydrae* [(en.hy’drae) N.L. gen. n *enhydrae*, pertaining to sea otters, *Enydra lutris nereis,* from which this bacterium was isolated]. Description is based on a single strain. Cells are variably Gram-positive, facultative anaerobic, non-motile, α-hemolytic, and PCR-positive for the *spaA* gene. Positive for arginine dihydrolase, beta galactosidase, alkaline phosphatase, ribose, lactose, L-arabinose, alanyl-phenylalanyl-proline-arylamidase, beta galactosidase, pyrogulutamic acid arylamidase, N-acetyl beta-glucosaminidase, glycyl-tryptophan arlamidase, and tagatose. Weakly positive for Voges Proskauer. Negative for oxidase, catalase, beta glucosidase, beta glucuronidase, alpha galactosidase, mannitol, sorbitol, trehalose, raffinose, saccharose, D arabitol, cyclodextrin, hydrolysis of hippurate, glycogen, pullulane, maltose, melibiose, melezitose, methyl-beta glucopyranoside, beta mannosidase, and urease. The major fatty acids are 16:0, 18:1 w8c, and 18:0. The bacterium can be stored at −80°C in 20% glycerol for long-term storage. The whole genome of UCD-4322-04 isolated from a clinically affected southern sea otter is accessible from GenBank (CP125807).

*Erysipelothrix rhusiopathiae ohloneorum* (oh.lo.ne.o’rum N.L. gen. pl. n. *ohloneorum,* pertaining to the Ohlone people who are native to the coastal area from which this bacterium was isolated). Cells are variably Gram-positive, facultative anaerobic, non-motile, α-hemolytic, and PCR-positive for the *spaA* gene. Positive for arginine dihydrolase, beta galactosidase, alkaline phosphatase, ribose, lactose, L-arabinose, alanyl-phenylalanyl-proline-arylamidase, beta galactosidase, pyrogulutamic acid arylamidase, N-acetyl beta-glucosaminidase, and glycyl-tryptophan arlamidase. Negative for oxidase, catalase, beta glucosidase, beta glucuronidase, alpha galactosidase, mannitol, sorbitol, trehalose, raffinose, saccharose, D arabitol, cyclodextrin, Voges Proskauer, hydrolysis of hippurate, glycogen, pullulane, maltose, melibiose, melezitose, methyl-beta glucopyranoside, tagatose, beta mannosidase, and urease. The major fatty acids are 16:0, 18:1 w9c, 20:4 w6, 9, 12, 15c, and 18:0. The bacterium can be stored at −80°C in 20% glycerol for long-term storage. The whole genome of UCD-4724-06 isolated from a clinically affected southern sea otter is accessible from GenBank (CP125808).

## References

[ref1] Auch A. F. Von Jan M. Klenk H. P. Göker M. (2010). Digital DNA-DNA hybridization for microbial species delineation by means of genome-to-genome sequence comparison. Stand. Genomic Sci. 2, 117–134. doi: 10.4056/sigs.531120, PMID: 21304684 PMC3035253

[ref2] Aziz R. K. Bartels D. Best A. A. DeJongh M. Disz T. Edwards R. A.. (2008). The RAST server: rapid annotations using subsystems technology. BMC Genomics 9:75. doi: 10.1186/1471-2164-9-75, PMID: 18261238 PMC2265698

[ref3] Bannantine J. P. Conde C. Bayles D. O. Branger M. Biet F. (2020). Genetic diversity among *Mycobacterium avium* subspecies revealed by analysis of complete genome sequences. Front. Microbiol. 11:1701. doi: 10.3389/fmicb.2020.01701, PMID: 32849358 PMC7426613

[ref4] Bartlett G. Smith W. Dominik C. Batac F. Dodd E. Byrne B. A.. (2016). Prevalence, pathology, and risk factors associated with *Streptococcus phocae* infection in southern sea otters (*Enhydra lutris nereis*), 2004-10. J. Wildl. Dis. 52, 1–9. doi: 10.7589/2015-02-048, PMID: 26555115

[ref5] Baum B. R. (1989). PHYLIP: phylogeny inference package. Version 3.2. Joel Felsenstein. Q. Rev. Biol. 64, 539–541. doi: 10.1086/416571

[ref6] Bolger A. M. Lohse M. Usadel B. (2014). Trimmomatic: a flexible trimmer for Illumina sequence data. Bioinformatics 30, 2114–2120. doi: 10.1093/bioinformatics/btu170, PMID: 24695404 PMC4103590

[ref7] Boseret G. Saegerman C. Mainil J. Jauniaux T. (2012). *Water-borne emerging zoonoses case report on erysipelas (Erysipelothrix rhusiopathiae) in harbour porpoises (Phocoena phocoena) and harbour seal (Phoca vitulina)*. Available at: https://orbi.uliege.be/handle/2268/128664. (Accessed Mar 28, 2022).

[ref8] Cabrera-García A. I. Müller F. Rödler F. S. Traub F. Heilmann R. M. (2020). Infective endocarditis due to *Erysipelothrix rhusiopathiae* in a dog – a case report. BMC Vet. Res. 16:328. doi: 10.1186/s12917-020-02546-6, PMID: 32912219 PMC7488128

[ref9] Ceccolini M. E. Wessels M. Macgregor S. K. Deaville R. Perkins M. Jepson P. D.. (2021). Systemic *Erysipelothrix rhusiopathiae* in seven free-ranging delphinids stranded in England and Wales. Dis. Aquat. Org. 145, 173–184. doi: 10.3354/dao03609, PMID: 34263732

[ref10] Chinn S. M. Miller M. A. Tinker M. T. Staedler M. M. Batac F. I. Dodd E. M.. (2016). The high cost of motherhood: end-lactation syndrome in southern sea otters (*Enhydra lutris nereis*) on the Central California coast, USA. J. Wildl. Dis. 52, 307–318. doi: 10.7589/2015-06-158, PMID: 26967137

[ref11] Choo S. W. Rishik S. Wee W. Y. (2020). Comparative genome analyses of *Mycobacteroides immunogenum* reveals two potential novel subspecies. Microb. Genom. 6:mgen000495. doi: 10.1099/mgen.0.000495 33295861 PMC8116688

[ref12] Ciufo S. Kannan S. Sharma S. Badretdin A. Clark K. Turner S.. (2018). Using average nucleotide identity to improve taxonomic assignments in prokaryotic genomes at the NCBI. Int. J. Syst. Evol. Microbiol. 68, 2386–2392. doi: 10.1099/ijsem.0.002809, PMID: 29792589 PMC6978984

[ref13] CLSI (2017). Methods for antimicrobial susceptibility testing of infrequently isolated or fastidious bacteria isolated from animals. 1st Edn. Wayne, PA: Clinical and Laboratory Standards Institute, CLSI Supplement VET06.

[ref14] Connell R. Moynihan I. W. Frank J. F. (1952). Studies of swine erysipelas. i. Literature review and survey of *Erysipelothrix rhusiopathiae* infection in Canada. Can. J. Comp. Med. Vet. Sci. 16, 104–128. PMID: 17648552 PMC1791404

[ref15] Danecek P. Bonfield J. K. Liddle J. Marshall J. Ohan V. Pollard M. O.. (2021). Twelve years of SAMtools and BCFtools. GigaScience 10:giab008. doi: 10.1093/gigascience/giab008, PMID: 33590861 PMC7931819

[ref16] De Coster W. D’Hert S. Schultz D. T. Cruts M. Van Broeckhoven C. (2018). NanoPack: visualizing and processing long-read sequencing data. Bioinform. Oxf. Engl. 34, 2666–2669. doi: 10.1093/bioinformatics/bty149, PMID: 29547981 PMC6061794

[ref17] Dudek N. K. Switzer A. D. Costello E. K. Murray M. J. Tomoleoni J. A. Staedler M. M.. (2022). Characterizing the oral and distal gut microbiota of the threatened southern sea otter (*Enhydra lutris nereis*) to enhance conservation practice. Conserv. Sci. Pract. 4:e12640. doi: 10.1111/csp2.12640, PMID: 35382031 PMC8979051

[ref18] Dunbar S. A. Clarridge J. E. (2000). Potential errors in recognition of *Erysipelothrix rhusiopathiae*. J. Clin. Microbiol. 38, 1302–1304. doi: 10.1128/JCM.38.3.1302-1304.2000, PMID: 10699048 PMC88613

[ref19] Edgar R. C. (2004). MUSCLE: multiple sequence alignment with high accuracy and high throughput. Nucleic Acids Res. 32, 1792–1797. doi: 10.1093/nar/gkh340, PMID: 15034147 PMC390337

[ref20] Eisenberg T. Mühldorfer K. Erhard M. Fawzy A. Kehm S. Ewers C.. (2022). *Erysipelothrix anatis* sp. nov., *Erysipelothrix aquatica* sp. nov. and *Erysipelothrix urinaevulpis* sp. nov., three novel species of the genus, and emended description of *Erysipelothrix*. Int. J. Syst. Evol. Microbiol. 72:005454. doi: 10.1099/ijsem.0.005454 35776769

[ref21] Elvy J. Hanspal I. Simcock P. (2008). A case of *Erysipelothrix rhusiopathiae* causing bilateral endogenous endophthalmitis. J. Clin. Pathol. 61, 1223–1224. doi: 10.1136/jcp.2008.060251 18818268

[ref22] Eriksson H. Jansson D. S. Johansson K. E. Båverud V. Chirico J. Aspán A. (2009). Characterization of *Erysipelothrix rhusiopathiae* isolates from poultry, pigs, emus, the poultry red mite and other animals. Vet. Microbiol. 137, 98–104. doi: 10.1016/j.vetmic.2008.12.016, PMID: 19193500

[ref23] Fidalgo S. G. Longbottom C. J. Riley T. V. (2002). Susceptibility of *Erysipelothrix rhusiopathiae* to antimicrobial agents and home disinfectants. Pathology (Phila.) 34, 462–465. doi: 10.1080/0031302021000009405 12408347

[ref24] Fiorito C. D. Bentancor A. Lombardo D. Bertellotti M. (2016). *Erysipelothrix rhusiopathiae* isolated from gull-inflicted wounds in southern right whale calves. Dis. Aquat. Org. 121, 67–73. doi: 10.3354/dao03041, PMID: 27596861

[ref25] Forde T. Biek R. Zadoks R. Workentine M. L. De Buck J. Kutz S.. (2016). Genomic analysis of the multi-host pathogen *Erysipelothrix rhusiopathiae* reveals extensive recombination as well as the existence of three generalist clades with wide geographic distribution. BMC Genomics 17:461. doi: 10.1186/s12864-016-2643-0, PMID: 27301771 PMC4906694

[ref26] Gorby G. L. Peacock J. E. (1988). *Erysipelothrix rhusiopathiae* endocarditis: microbiologic, epidemiologic, and clinical features of an occupational disease. Rev. Infect. Dis. 10, 317–325. doi: 10.1093/clinids/10.2.317, PMID: 3287562

[ref27] Goris J. Konstantinidis K. T. Klappenbach J. A. Coenye T. Vandamme P. Tiedje J. M. (2007). DNA-DNA hybridization values and their relationship to whole-genome sequence similarities. Int. J. Syst. Evol. Microbiol. 57, 81–91. doi: 10.1099/ijs.0.64483-0 17220447

[ref28] Grazziotin A. L. Vidal N. M. Hoepers P. G. Reis T. F. M. Mesa D. Caron L. F.. (2021). Comparative genomics of a novel clade shed light on the evolution of the genus *Erysipelothrix* and characterise an emerging species. Sci. Rep. 11, 1–12. doi: 10.1038/s41598-021-82959-x, PMID: 33564084 PMC7873064

[ref29] Grieco M. H. Sheldon C. (1970). Erysipelothrix rhusiopathiae. Ann. N. Y. Acad. Sci. 174, 523–532. doi: 10.1111/j.1749-6632.1970.tb45578.x 5278133

[ref30] Gunderson C. G. Martinello R. A. (2012). A systematic review of bacteremias in cellulitis and erysipelas. J. Infect. 64, 148–155. doi: 10.1016/j.jinf.2011.11.004, PMID: 22101078

[ref31] Habte D. Tilahun D. T. Tilahun T. (2021). Swine erysipelas; its epidemiology, diagnosis, treatment and control and preventive measures, comprehensive review. J. Clin. Epidemiol. Toxicol. 2, 1–7. doi: 10.47363/JCET/2020(2)115

[ref32] Higgins R. (2000). Bacteria and fungi of marine mammals: a review. Can. Vet. J. 41, 105–116. PMID: 10723596 PMC1476275

[ref33] IJsseldijk L. L. Begeman L. Duim B. Gröne A. MJL K. Klijnstra M. D.. (2023). Harbor porpoise deaths associated with *Erysipelothrix rhusiopathiae*, the Netherlands, 2021. Emerg. Infect. Dis. 29, 835–838. doi: 10.3201/eid2904.221698 36958025 PMC10045706

[ref34] Jain C. Rodriguez-R L. M. Phillippy A. M. Konstantinidis K. T. Aluru S. (2018). High throughput ANI analysis of 90K prokaryotic genomes reveals clear species boundaries. Nat. Commun. 9:5114. doi: 10.1038/s41467-018-07641-9 30504855 PMC6269478

[ref35] Janßen T. Voss M. Kühl M. Semmler T. Philipp H. C. Ewers C. (2015). A combinational approach of multilocus sequence typing and other molecular typing methods in unravelling the epidemiology of *Erysipelothrix rhusiopathiae* strains from poultry and mammals. Vet. Res. 46:84. doi: 10.1186/s13567-015-0216-x, PMID: 26198736 PMC4509749

[ref36] Jean S. Lainhart W. Yarbrough M. L. (2019). The brief case: Erysipelothrix bacteremia and endocarditis in a 59-year-old immunocompromised male on chronic high-dose steroids. J. Clin. Microbiol. 57, e02031–e02018. doi: 10.1128/JCM.02032-18 31127013 PMC6535599

[ref37] Katoh K. Asimenos G. Toh H. (2009). Multiple alignment of DNA sequences with MAFFT. Methods Mol. Biol. 537, 39–64. doi: 10.1007/978-1-59745-251-9_3 19378139

[ref38] Kearse M. Moir R. Wilson A. Stones-Havas S. Cheung M. Sturrock S.. (2012). Geneious basic: an integrated and extendable desktop software platform for the organization and analysis of sequence data. Bioinformatics 28, 1647–1649. doi: 10.1093/bioinformatics/bts199, PMID: 22543367 PMC3371832

[ref39] Kinsel M. J. Boehm J. R. Harris B. Murnane R. D. (1997). Fatal *Erysipelothrix rhusiopathiae* septicemia in a captive Pacific white-sided dolphin (*Lagenorhyncus obliquidens*). J. Zoo Wildl. Med. 28, 494–497. PMID: 9523647

[ref40] Konstantinidis K. T. Tiedje J. M. (2005a). Genomic insights that advance the species definition for prokaryotes. Proc. Natl. Acad. Sci. U. S. A. 102, 2567–2572. doi: 10.1073/pnas.0409727102, PMID: 15701695 PMC549018

[ref41] Konstantinidis K. T. Tiedje J. M. (2005b). Towards a genome-based taxonomy for prokaryotes. J. Bacteriol. 187, 6258–6264. doi: 10.1128/JB.187.18.6258-6264.2005, PMID: 16159757 PMC1236649

[ref42] Koren S. Walenz B. P. Berlin K. Miller J. R. Bergman N. H. Phillippy A. M. (2017). Canu: scalable and accurate long-read assembly via adaptive k-mer weighting and repeat separation. Genome Res. 27, 722–736. doi: 10.1101/gr.215087.116, PMID: 28298431 PMC5411767

[ref43] Kumar S. Stecher G. Tamura K. (2016). Mega7: molecular evolutionary genetics analysis version 7.0 for bigger datasets. Mol. Biol. Evol. 33, 1870–1874. doi: 10.1093/molbev/msw054, PMID: 27004904 PMC8210823

[ref44] Kutz S. Bollinger T. Branigan M. Checkley S. Davison T. Dumond M.. (2015). *Erysipelothrix rhusiopathiae* associated with recent widespread muskox mortalities in the Canadian Arctic. Can. Vet. J. 56, 560–563. PMID: 26028673 PMC4431149

[ref45] Li H. (2021). New strategies to improve minimap2 alignment accuracy. Bioinformatics 37, 4572–4574. doi: 10.1093/bioinformatics/btab705, PMID: 34623391 PMC8652018

[ref46] Malik Y. S. Arun Prince Milton A. Ghatak S. Ghosh S. (2021). “Avian Erysipelas” in Role of birds in transmitting zoonotic pathogens. eds. Malik Y. S. Arun Prince Milton A. Ghatak S. Ghosh S. (Singapore: Springer), 163–170.

[ref47] Marshall K. R. Walton S. A. Boyd M. Bishop B. Wellehan J. Craft W.. (2019). Erysipeloid lesions caused by *Erysipelothrix rhusiopathiae* in a dog: clinical and histopathological findings, molecular diagnosis and treatment. Vet. Dermatol. 30, 434–e134. doi: 10.1111/vde.12773, PMID: 31364229

[ref48] McLauchlin J. Riegel P. (2012). “17- Coryneform bacteria, Listeria and Erysipelothrix: diphtheria; listeriosis; erysipeloid” in Medical Microbiology. eds. Greenwood D. Barer M. Slack R. Irving W.. 18th ed (Edinburgh: Churchill Livingstone), 199–210.

[ref49] Meier-Kolthoff J. P. Auch A. F. Klenk H. P. Göker M. (2013). Genome sequence-based species delimitation with confidence intervals and improved distance functions. BMC Bioinformatics 14:60. doi: 10.1186/1471-2105-14-60, PMID: 23432962 PMC3665452

[ref50] Meier-Kolthoff J. P. Carbasse J. S. Peinado-Olarte R. L. Göker M. (2021). TYGS and LPSN: a database tandem for fast and reliable genome-based classification and nomenclature of prokaryotes. Nucleic Acids Res. 50, D801–D807. doi: 10.1093/nar/gkab902 PMC8728197 34634793

[ref51] Meier-Kolthoff J. P. Hahnke R. L. Petersen J. Scheuner C. Michael V. Fiebig A.. (2014). Complete genome sequence of DSM 30083T, the type strain (U5/41T) of Escherichia coli, and a proposal for delineating subspecies in microbial taxonomy. Stand. Genomic Sci. 9:2. doi: 10.1186/1944-3277-9-2, PMID: 25780495 PMC4334874

[ref52] Melero M. Rubio-Guerri C. Crespo J. Arbelo M. Vela A. García-Párraga D.. (2011). First case of erysipelas in a free-ranging bottlenose dolphin (*Tursiops truncatus*) stranded in the Mediterranean Sea. Dis. Aquat. Org. 97, 167–170. doi: 10.3354/dao02412, PMID: 22303633

[ref53] Miller M. A. Moriarty M. E. Duignan P. J. Zabka T. S. Dodd E. Batac F. I.. (2021). Clinical signs and pathology associated with domoic acid toxicosis in southern sea otters (*Enhydra lutris nereis*). Front. Mar. Sci. 8:5501. doi: 10.3389/fmars.2021.585501

[ref54] Miller M. A. Moriarty M. E. Henkel L. Tinker M. T. Burgess T. L. Batac F. I.. (2020). Predators, disease, and environmental change in the nearshore ecosystem: mortality in southern sea otters (*Enhydra lutris* nereis) from 1998–2012. Front. Mar. Sci. 7:582. doi: 10.3389/fmars.2020.00582/full

[ref55] Nakato H. Shinomaya K. Mikawa H. (1985). Experimental arteritis in young rats induced by *Erysipelothrix rhusiopathiae*. Exp. Pathol. 28, 97–104. doi: 10.1016/S0232-1513(85)80020-7, PMID: 3930281

[ref56] Opriessnig T. Forde T. Shimoji Y. (2020). *Erysipelothrix* spp.: past, present, and future directions in vaccine research. Front Vet Sci 7:174. doi: 10.3389/fvets.2020.00174, PMID: 32351978 PMC7174600

[ref57] Opriessnig T. Shen H. G. Bender J. S. Boehm J. R. Halbur P. G. (2013). *Erysipelothrix rhusiopathiae* isolates recovered from fish, a harbour seal (*Phoca vitulina*) and the marine environment are capable of inducing characteristic cutaneous lesions in pigs. J. Comp. Pathol. 148, 365–372. doi: 10.1016/j.jcpa.2012.08.004, PMID: 23083834

[ref58] Pearce M. E. Langridge G. C. Lauer A. C. Grant K. Maiden M. C. J. Chattaway M. A. (2021). An evaluation of the species and subspecies of the genus *Salmonella* with whole genome sequence data: proposal of type strains and epithets for novel *S. enterica* subspecies VII, VIII, IX, X and XI. Genomics 113, 3152–3162. doi: 10.1016/j.ygeno.2021.07.003, PMID: 34242711 PMC8426187

[ref59] Pomaranski E. K. Griffin M. J. Camus A. C. Armwood A. R. Shelley J. Waldbieser G. C.. (2020). Description of *Erysipelothrix piscisicarius* sp. nov., an emergent fish pathogen, and assessment of virulence using a tiger barb (*Puntigrus tetrazona*) infection model. Int. J. Syst. Evol. Microbiol. 70, 857–867. doi: 10.1099/ijsem.0.003838 31682217

[ref60] Pomaranski E. K. Reichley S. R. Yanong R. Shelley J. Pouder D. B. Wolf J. C.. (2018). Characterization of spa C-type *Erysipelothrix* sp. isolates causing systemic disease in ornamental fish. J. Fish Dis. 41, 49–60. doi: 10.1111/jfd.12673 28708262

[ref61] Prod’Hom G. Bille J. (2010). “Aerobic gram-positive bacilli” in Infectious diseases. eds. Cohen J. Opal S. M. Powderly W. G.. 3rd ed (London: Mosby), 1660–1675.

[ref62] Reboli A. C. Farrar W. E. (1989). *Erysipelothrix rhusiopathiae*: an occupational pathogen. Clin. Microbiol. Rev. 2, 354–359. doi: 10.1128/CMR.2.4.354, PMID: 2680056 PMC358129

[ref63] Renz H. Gentz U. Schmidt A. Dapper T. Nain M. Gemsa D. (1989). Activation of macrophages in an experimental rat model of arthritis induced by *Erysipelothrix rhusiopathiae* infection. Infect. Immun. 57, 3172–3180. doi: 10.1128/iai.57.10.3172-3180.1989, PMID: 2789193 PMC260786

[ref64] Richter M. Rosselló-Móra R. (2009). Shifting the genomic gold standard for the prokaryotic species definition. Proc. Natl. Acad. Sci. U. S. A. 106, 19126–19131. doi: 10.1073/pnas.0906412106, PMID: 19855009 PMC2776425

[ref65] Salzberg S. L. Delcher A. L. Kasif S. White O. (1998). Microbial gene identification using interpolated Markov models. Nucleic Acids Res. 26, 544–548. doi: 10.1093/nar/26.2.544, PMID: 9421513 PMC147303

[ref66] Seahorn T. L. Brumbaugh G. W. Carter G. K. Wood R. L. (1989). *Erysipelothrix rhusiopathiae* bacteremia in a horse. Cornell Vet. 79, 151–156. PMID: 2924578

[ref67] Shen H. G. Bender J. S. Opriessnig T. (2010). Identification of surface protective antigen (spa) types in *Erysipelothrix* reference strains and diagnostic samples by spa multiplex real-time and conventional PCR assays. J. Appl. Microbiol. 109, 1227–1233. doi: 10.1111/j.1365-2672.2010.04746.x 20477888

[ref68] Shimazaki Y. Gamoh K. Imada Y. Makie H. Kanzaki M. Takahashi T. (2005). Detection of antibodies to *Erysipelothrix* in stray dogs in Japan. Acta Vet. Scand. 46, 159–161. doi: 10.1186/1751-0147-46-159, PMID: 16261928 PMC1624815

[ref69] Shinomiya K. Nakato H. (1985). An experimental model of arteritis: periarteritis induced by *Erysipelothrix rhusiopathiae* in young rats. Int. J. Tissue React. 7, 267–271. PMID: 4066201

[ref70] Suer L. D. Vedros N. A. (1988). *Erysipelothrix rhusiopathiae. I. Isolation and characterization from pinnipeds and bitelabrasion wounds in humans*.

[ref71] Suer L. D. Vedros N. A. Schroeder J. P. Dunn J. L. (1988). *Erysipelothrix rhusiopathiae*. II. Enzyme immunoassay of sera from wild and captive marine mammals. Dis. Aquat. Org. 5, 7–13. doi: 10.3354/dao005007

[ref72] Tamura K. Nei M. (1993). Estimation of the number of nucleotide substitutions in the control region of mitochondrial DNA in humans and chimpanzees. Mol. Biol. Evol. 10, 512–526. doi: 10.1093/oxfordjournals.molbev.a040023, PMID: 8336541

[ref73] Tatusova T. DiCuccio M. Badretdin A. Chetvernin V. Nawrocki E. P. Zaslavsky L.. (2016). NCBI prokaryotic genome annotation pipeline. Nucleic Acids Res. 44, 6614–6624. doi: 10.1093/nar/gkw569 27342282 PMC5001611

[ref74] To H Someno S. Nagai S. Koyama T. Nagano T. (2010). Immunization with truncated recombinant protein Spa C of *Erysipelothrix rhusiopathiae* strain 715 serovar 18 confers protective immunity against challenge with various serovars. Clin. Vacc. Immunol. 17, 1991–1997. doi: 10.1128/CVI.00213-10, PMID: 20926696 PMC3008202

[ref75] To H. Nagai S. (2007). Genetic and antigenic diversity of the surface protective antigen proteins of *Erysipelothrix rhusiopathiae*. Clin. Vaccine Immunol. 14, 813–820. doi: 10.1128/CVI.00099-07, PMID: 17475766 PMC1951066

[ref76] Venditti M. Gelfusa V. Tarasi A. Brandimarte C. Serra P. (1990). Antimicrobial susceptibilities of *Erysipelothrix rhusiopathiae*. Antimicrob. Agents Chemother. 34, 2038–2040. doi: 10.1128/AAC.34.10.2038, PMID: 2291674 PMC171989

[ref77] Veraldi S. Girgenti V. Dassoni F. Gianotti R. (2009). Erysipeloid: a review. Clin. Exp. Dermatol. 34, 859–862. doi: 10.1111/j.1365-2230.2009.03444.x, PMID: 19663854

[ref78] Walker B. J. Abeel T. Shea T. Priest M. Abouelliel A. Sakthikumar S.. (2014). Pilon: an integrated tool for comprehensive microbial variant detection and genome assembly improvement. PLoS One 9:e112963. doi: 10.1371/journal.pone.0112963, PMID: 25409509 PMC4237348

[ref79] Wang Q. Chang B. J. Riley T. V. (2010). Erysipelothrix rhusiopathiae. Vet. Microbiol. 140, 405–417. doi: 10.1016/j.vetmic.2009.08.012 19733019

[ref80] Wattrang E. Eriksson H. Jinnerot T. Persson M. Bagge E. Söderlund R.. (2020). Immune responses upon experimental *Erysipelothrix rhusiopathiae* infection of naïve and vaccinated chickens. Vet. Res. 51:114. doi: 10.1186/s13567-020-00830-9, PMID: 32928307 PMC7488726

[ref81] Williams T. D. Allen D. D. Groff J. M. Glass R. L. (1992). An analysis of California Sea otter (*Enhydra lutris*) pelage and integument. Mar. Mamm. Sci. 8, 1–18. doi: 10.1111/j.1748-7692.1992.tb00120.x

[ref82] Zhong J. Medvecky M. Tornos J. Clessin A. Gantelet H. Gamble A.. (2022). Genomic characterisation of a novel species of Erysipelothrix associated with mortalities among endangered seabirds. bioRxiv 2022:497316. doi: 10.1101/2022.06.23.497316v1 38359084

[ref83] Zhu W. Wu C. Kang C. Cai C. Wang Y. Li J.. (2018). Evaluation of the protective efficacy of four newly identified surface proteins of *Erysipelothrix rhusiopathiae*. Vaccine 36, 8079–8083. doi: 10.1016/j.vaccine.2018.10.071, PMID: 30446176

