# Supplementary material for: Pathology, microbiology, and genetic diversity associated with Erysipelothrix rhusiopathiae and novel Erysipelothrix spp. infections in southern sea otters (Enhydra lutris nereis)
Source: Front Microbiol. 2024 Feb 1;14:1303235. doi: 10.3389/fmicb.2023.1303235 (PMC10867225; doi:10.3389/fmicb.2023.1303235)
Supplement: Supplementary file 1 [file Data_Sheet_1.docx]

**Supplemental Table 1.** Primer sequences for surface protective antigen typing of *Erysipelothrix* sp. isolates from necropsied southern sea otters (*Enhydra lutris nereis*) used in this study (32).

| Gene | Size (bp) | Primer Sequence (5’-3’) |
| --- | --- | --- |
| *spaA* | 103 | F: CAGCAATGCCACTACAAACAGC  R: CCTGTCCCAGGTAAAACTGGG |
| *spaB* | 112 | F: CAGCAATGCCACTACAAACAGC  R: GCATCGTCATCAATTCTGGGTAG |
| *spaC* | 96 | F: CAGCAATGCCACTACAAACAGC  R: CAGGTATGATTGGAAGTGTTCCG |

**Supplemental Table 2.** PCR primers from Janβen et al. and Pomaranski et al. (10, 54) used for multi-locus sequence analysis of *Erysipelothrix* spp. isolates recovered from necropsied southern sea otters (*Enhydra lutris nereis*).

| Gene Name | Gene Product | Sequence (5’ – 3’) | Amplicon Size (bp) | Accession Number |
| --- | --- | --- | --- | --- |
| *galk* | Galactokinase | F: TATTCCTAATGGAGCGGG  R: AATCGCAATCGCACATCC | 685 | MN215495  MN215498 |
| *gpsA* | Glycerol-3-phosphate-dehydrogenase | F: AGTTATGATGTGGGGACG  R: TAGCTGTAACGACGAGATCG | 540 | MN215491  -MN215494 |
| *ldhA* | D-lactate dehydrogenase | F: AACGGATATGAAGCTGTTGCC  R: AAGAACATCCAGTCCAACAGC | 648 | MN215499  -  MN215502 |
| *prsA* | Ribose-phosphate-pyrophosphokinase | F: ACAAGTTCACCAGTAAGTG  R: AGAGTGTACTTACAGGAGT | 763 | MN215503  -  MN215506 |
| *pta* | Phosphate acetyl-transferase | F: TGCTGCAGTACGTTTAGC  R: AGACACGTGCATTACCTG | 704 | MN215507  -  MN215510 |
| *purA* | Adenylosuccinate synthetase | F: GATGTTTATGAGGAAGCGC  R: AACGCATTGATTGTTGCCC | 736 | MN215511  -  MN215514 |
| *recA* | Recombinase A | F: TTCGGTAGAATAATCTCGCG  R: TGCTATTAGTTCAGGGTCG | 876 | MN215515  -  MN215518 |
| *gyrB* | Gyrase B | F: GTGGAACGCATGAAGATGGATTT  R: CAGCATATTCAGCATTTGCCTGA | 853 | MN213145-  MN213148 |

**Supplemental Table 3.** Isolate identification, source, and *spa-*type of *Erysipelothrix* spp. isolates used for MLSA and dendrogram analysis. All isolates from Pomaranski et al. (2).

| Isolate | Species | Bacteria Isolated | *spa* | Location |
| --- | --- | --- | --- | --- |
| GXBY-1 | Porcine | *E. rhusiopathiae* | A | Acc: NZ_CP014861 |
| SY1027 | Porcine | *E. rhusiopathiae* | A | Acc: NC_021354 |
| Fujisawa | Porcine | *E. rhusiopathiae* | A | Acc: NC_015601 |
| CAHFS4 | Caprine - liver | *E. rhusiopathiae* | A | CAHFS UC Davis |
| CAHFS1 | Chicken - liver | *E. rhusiopathiae* | A | CAHFS UC Davis |
| CAHFS2 | Ovine - liver | *E. rhusiopathiae* | A | CAHFS UC Davis |
| H1T1 | Herring | *E. rhusiopathiae* | A | Shedd Aquarium |
| 10506 | Dolphin - blood | *E. rhusiopathiae* | A | Shedd Aquarium |
| 6567 | Beluga – blood | *E. rhusiopathiae* | A | Shedd Aquarium |
| C1T0A | Feed (capelin) | *E. rhusiopathiae* | A | Shedd Aquarium |
| 9301985 | Elephant seal – lung | *E. rhusiopathiae* | A | VMTH UC Davis |
| C1T0B | Feed (capelin) | *E. rhusiopathiae* | A | Shedd Aquarium |
| CAP2 | Feed (capelin) | *E. rhusiopathiae* | A | Shedd Aquarium |
| 262 | Dolphin – spleen | *E. rhusiopathiae* | B | Shedd Aquarium |
| 10792 | Beluga – blood | *E. rhusiopathiae* | B | Shedd Aquarium |
| S4T0 | Feed (squid) | *E. rhusiopathiae* | B | Shedd Aquarium |
| 7122 | Beluga – blood | *E. rhusiopathiae* | B | Shedd Aquarium |
| DF-KriB | Dolphin – feces | *E. rhusiopathiae* | B | Shedd Aquarium |
| S1T0 | Feed – squid | *E. rhusiopathiae* | B | Shedd Aquarium |
| Strain 715 | Porcine | *Erysipelothrix* sp. | C | PRJNA288715 |
| 14089A | Jewel tetra- kidney | *E. piscisicarius* | C | Shedd Aquarium |
| UGA21756 | Ornamental Fish | *E. piscisicarius* | C | University of Georgia |
| 15TAL0474 | Ornamental Fish | *E. piscisicarius* | C | Florida Farm |
| 14TAL259A | Ornamental Fish | *E. piscisicarius* | C | Florida Farm |
| 9711 | Rasbora – kidney | *E. piscisicarius* | C | Shedd Aquarium |
| 14TAL261U2 | Ornamental Fish | *E. piscisicarius* | C | Florida Farm |
| 14TAL056U8 | Ornamental Fish | *E. piscisicarius* | C | Florida Farm |
| 15TAL055K2 | Ornamental Fish | *E. piscisicarius* | C | Florida Farm |
| 15TAL055U1 | Ornamental Fish | *E. piscisicarius* | C | Florida Farm |
| 14TAL259B | Ornamental Fish | *E. piscisicarius* | C | Florida Farm |
| 14TABl259C | Ornamental Fish | *E. piscisicarius* | C | Florida Farm |
| 14TAL260U1 | Ornamental Fish | *E. piscisicarius* | C | Florida Farm |
| 15TAL056K5 | Ornamental Fish | *E. piscisicarius* | C | Florida Farm |
| 15TAL056U3 | Ornamental Fish | *E. piscisicarius* | C | Florida Farm |

**Supplemental Table 4.** Minimum inhibitory concentrations (μg/mL) of antimicrobial drugs on *Erysipelothrix* spp. isolates from necropsied southern sea otters (*Enhydra lutris nereis*). Susceptibility interpretation following Clinical Laboratory Standards Institute breakpoints of *Erysipelothrix rhusiopathiae* for human isolates (29) (S=susceptible, I=intermediate, R=resistant, remaining not determined as breakpoints not present in CLSI reference). Control *Streptococcus pneumoniae* ATCC® 49619 within CLSI reference ranges.

|  | **4322-04** | **4724-06** | **3111-98** | **5818-10** | **6640-12** | **ATCC 49619** |
| --- | --- | --- | --- | --- | --- | --- |
|  | *E. enhydrae* sp. nov. | *E. rhusiopathiae ohloneorum* ssp. nov. | *E.*  *rhusiopathiae* | *E. rhusiopathiae* | *E. rhusiopathiae* | *Streptococcus pneumoniae* |
| Enrofloxacin | ≤0.12 (S) | ≤0.12 (S) | ≤0.12 (S) | ≤0.12 (S) | ≤0.12 (S) | 1 |
| Gentamicin | >8 | >8 | >8 | >8 | >8 | 8 |
| Ceftiofur | ≤0.25 (S) | 0.50 (S) | ≤0.25 (S) | ≤0.25 (S) | ≤0.25 (S) | ≤0.25 |
| Neomycin | >32 | >32 | >32 | >32 | >32 | 32 |
| Erythromycin | ≤0.12 (S) | ≤0.12 (S) | ≤0.12 (S) | ≤0.12 (S) | ≤0.12 (S) | ≤0.12 |
| Oxytetracycline | ≤0.25 | 0.5 | 0.5 | 0.5 | ≤0.25 | ≤0.25 |
| Tetracycline | ≤0.25 | 0.5 | 0.5 | 0.5 | ≤0.25 | ≤0.25 |
| Amoxicillin | ≤0.25 | ≤0.25 | ≤0.25 | ≤0.25 | ≤0.25 | ≤0.25 |
| Spectinomycin | ≤8 | ≤8 | ≤8 | ≤8 | ≤8 | 16 |
| Sulphadimethoxine | >256 | >256 | >256 | >256 | 256 | ≤32 |
| Trimethoprim/ sulfamethoxazole | 2/38 | >2/38 | >2/38 | >2/38 | >2/38 | ≤0.5/9.5 |
| Florfenicol | 4 | 8 | 8 | 4 | 4 | ≤1 |
| Sulphathiazole | >256 | >256 | >256 | >256 | >256 | ≤32 |
| Penicillin | ≤0.06 (S) | ≤0.06 (S) | ≤0.06 (S) | ≤0.06 (S) | ≤0.06 (S) | 0.5 |
| Streptomycin | ≤8 | ≤8 | ≤8 | ≤8 | 32 | 16 |
| Novobiocin | >4 | >4 | >4 | >4 | >4 | ≤0.5 |
| Tylosin tartrate | <2.5 | <2.5 | <2.5 | <2.5 | <2.5 | ≤2.5 |
| Clindamycin | <0.5 (S/I) | <0.5 (S/I) | <0.5 (S/I) | <0.5 (S/I) | <0.5 (S/I) | ≤0.5 |

**Supplemental Table 5.** Phenotypic properties of two novel *Erysipelothrix* spp. strains isolated from southern sea otters (SSO; *Enhydra lutris nereis*), one *E. rhusiopathiae* isolate from SSO (7692-15), four *E. rhusiopathiae* isolates, and one *E. piscisicarius* isolate.

|  | SSO 4322-04 | SSO 4724-06 | SSO 7692-15 | 6567 | H1T1 |  | 7122 | 7155 | 15TAL056U3 |
| --- | --- | --- | --- | --- | --- | --- | --- | --- | --- |
| *spa* | A | A | A | A | A |  | B | B | C |
| Test/Reaction | *Erysipelothrix enhydrae* sp. nov. | *E. rhusiopathiae ohloneorum* ssp. nov. | *E. rhusiopathiae* | *E. rhusiopathiae* | *E. rhusiopathiae* |  | *E. rhusiopathiae* | *E. rhusiopathiae* | *E. piscisicarius* |
| Growth in SBA at 30°C | + | + | + | + | + |  | + | + | + |
| Growth in BHIP at 30°C | + | + | + | + | + |  | + | + | + |
| Hemolysis | α | α | α | α | α |  | α | α | α |
| Oxidase | - | - | - | - | - |  | - | - | - |
| Catalase | - | - | - | - | - |  | - | - | - |
| Arginine dihydrolase (ADH) | + | + | + | + | + |  | + | + | + |
| Beta glucosidase (βGLU) | - | - | - | - | - |  | - | - | - |
| Beta galactosidase (βGAR) | + | + | + | + | + |  | + | + | **-** |
| Beta glucuronidase (βGUR) | - | + | + | + | + |  | + | + | + |
| Alpha galactosidase (αGAL) | - | - | - | - | - |  | - | - | - |
| Alkaline phosphatase (PAL) | + | + | + | + | + |  | + | + | + |
| Ribose (RIB) | + | + | + | + | + |  | + | + | + |
| Mannitol (MAN) | - | - | - | - | - |  | - | - | - |
| Sorbitol (SOR) | - | - | - | - | - |  | - | - | - |
| Lactose (LAC) | + | + | + | + | + |  | + | + | **-** |
| Trehalose (TRE) | - | - | - | - | - |  | - | - | - |
| Raffinose (RAF) | - | - | - | - | - |  | - | - | - |
| Saccharose (SAC) | - | - | - | - | - |  | - | - | - |
| L-arabinose (LARA) | + | + | + | + | + |  | + | + | + |
| D arabitol (DARL) | - | - | - | - | - |  | - | - | - |
| Cyclodextrin (CDEX) | - | - | - | - | - |  | - | - | - |
| Voges Proskauer (VP) | w+ | - | - | - | - |  | - | - | - |
| Alanyl-phenylalanyl-proline-arylamidase (APPA) | + | + | + | + | + |  | + | + | + |
| Beta galactosidase (βGAL) | + | + | + | + | + |  | + | + | + |
| Pyrogulutamic acid arylamidase (PyrA) | + | + | + | + | + |  | + | + | + |
| N-acetyl beta-glucosaminidase (βNAG) | + | + | + | + | + |  | + | + | + |
| Glycyl-tryptophan arlamidase (GTA) | + | + | + | + | + |  | + | + | + |
| Hydrolysis of Hippurate (HIP) | - | - | - | - | - |  | - | - | - |
| Glycogen (GLYG) | - | - | - | - | - |  | - | - | - |
| Pullulane (PUL) | - | - | - | - | - |  | - | - | - |
| Maltose (MAL) | - | - | - | - | - |  | - | - | - |
| Melibiose (MEL) | - | - | - | - | - |  | - | - | - |
| Melezitose (MLZ) | - | - | - | - | - |  | - | - | - |
| Methyl-beta glucopyranoside (MBDG) | - | - | - | - | - |  | - | - | - |
| Tagatose (TAG) | + | - | - | - | - |  | - | - | - |
| Beta mannosidase (ΒMAN) | - | - | - | - | - |  | - | - | - |
| Urease (URE) | - | - | - | - | - |  | - | - | - |

**Supplemental Table 6.** Fatty acid methyl esters (FAME) compositions (%) of two novel *Erysipelothrix* spp. strains isolated from two necropsied southern sea otters (SSO: *Enhydra lutris nereis)*, three *E. rhusiopathiae* isolates from marine mammals, one *E. rhusiopathiae* isolate from a fish, and one *E. piscisicarius* isolate.

|  | 4322-04 (SSO) | 4724-06 (SSO) | 7692-15 (SSO) | 6567 (beluga) | 7122 (beluga) | 7155 (beluga) | H1T1 (fish) | 15TAL0643 (fish) |
| --- | --- | --- | --- | --- | --- | --- | --- | --- |
| FAME | *Erysipelothrix enhydrae* sp. nov. | *E. rhusiopathiae ohloneorum* ssp. nov. | *E. rhusiopathiae* | *E. rhusiopathiae* | *E. rhusiopathiae* | *E. rhusiopathiae* | *E. rhusiopathiae* | *E. piscisicarius* |
| 8:0 3OH | ND^a^ | ND | 1.08 | ND | ND | 0.35 | ND | ND |
| 14:0 | 3.53 | 1.69 | 1.73 | 1.66 | 1.71 | 0.23 | ND | ND |
| 15:1 w5c | 0.78 | 0.70 | ND | 0.89 | ND | 0.46 | ND | ND |
| 16:1 w9c | 2.62 | 0.73 | 1.20 | 1.25 | ND | ND | ND | ND |
| 16:0 | 29.9 | 31.4 | 30.6 | 28.5 | 32.3 | 29.0 | 41.01 | 37.97 |
| 17:1 iso w5c | ND | 2.08 | ND | ND | ND | ND | ND | ND |
| 17:1 w8c | 0.83 | ND | ND | ND | ND | ND | ND | ND |
| 18:1 w9c | 20.8 | 19.0 | 19.8 | 16.8 | 21.9 | 17.8 | 24.60 | 21.29 |
| 18:0 | 14.0 | 12.0 | 12.1 | 11.6 | 12.5 | 14.8 | 16.77 | 12.89 |
| 20:4 w6,9,12,15c | 8.93 | 12.2 | 12.8 | 19.9 | 10.8 | 25.7 | 5.38 | 10.70 |
| 20:0 iso | 0.76 | ND | ND | ND | ND | ND | ND | ND |
| Summed Feature 3^b^ | 4.68 | 7.25 | 6.60 | 6.42 | 5.94 | 3.02 | ND | ND |
| Summed Feature 5^c^ | 7.10 | 6.33 | 6.62 | 6.76 | 8.22 | 3.25 | 3.81 | 8.23 |
| Summed Feature 8^d^ | 5.98 | 6.61 | 7.41 | 6.21 | 6.61 | 4.39 | 8.43 | 8.91 |

^a^ND = Fatty acids not detected

^b^ The fatty acids 16:1 ω7c/16:1 ω6c, 16:1 ω6c/16:1 ω7c could not be separated from each other and together were considered Summed Feature 3

^c^ The fatty acids 18:0 ante/18:2 ω6, 9c, 18:2 ω6,9c/18:0 ante could not be separated from each other and were considered Summed Feature 5

^d^ The fatty acids 18:1 ω7c, 18:1 ω6c could not be separated from each other and together were considered Summed Feature 8

**Supplemental Figure 1.** Amino acid sequence alignment (ClustalW) of surface protective antigen proteins performed in Mega v7. SpaA-positive *Erysipelothrix*spp. isolates 4322-04 and 4724-06 from southern sea otters (*Enhydra lutris nereis*) were aligned with sixteen discrete *Erysipelothrix*spp. isolates representing *spaA, spaB*, and *spaC* isoforms (55). Residues conserved across multiple sequences are represented by a shared color.

**
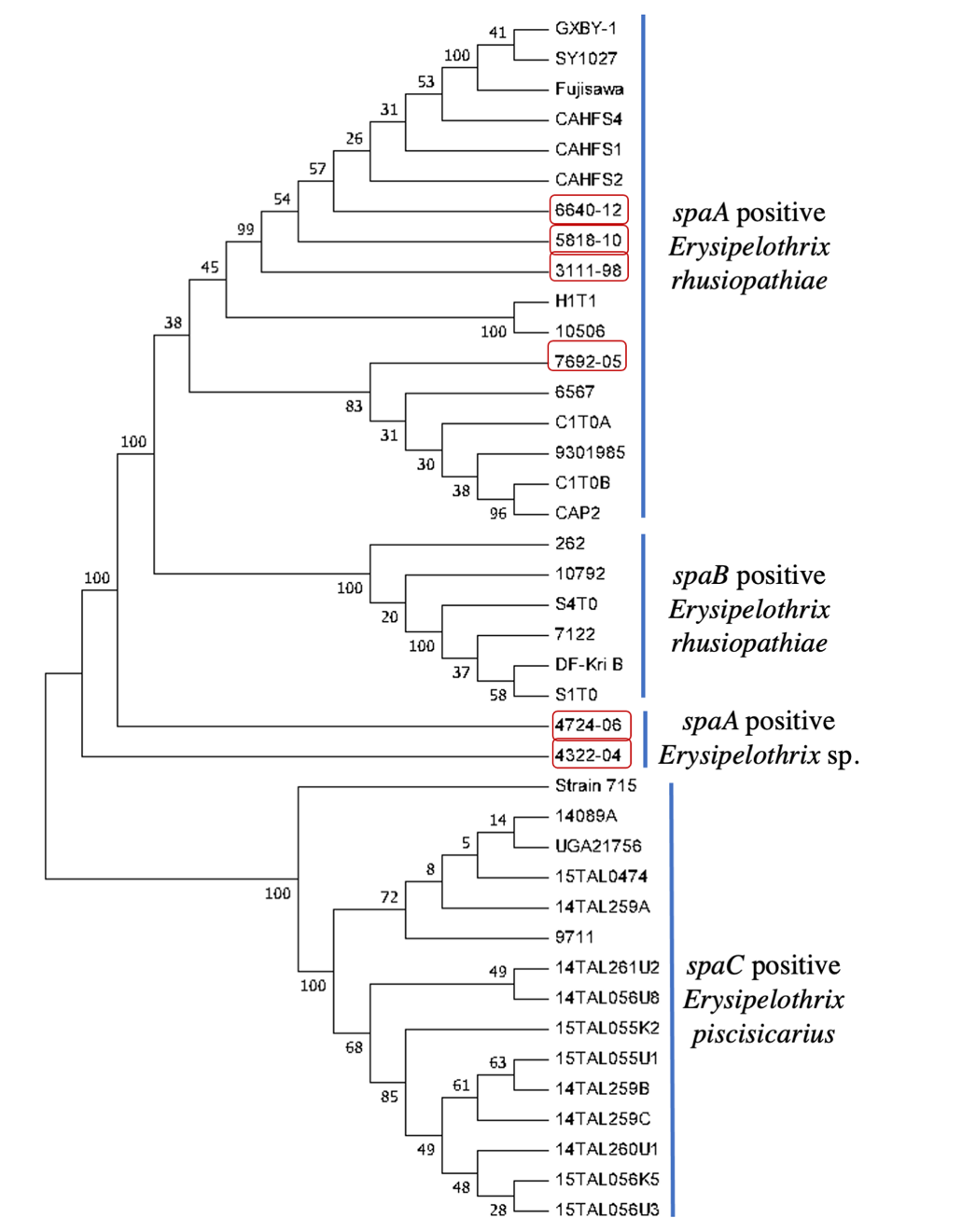
**

**Supplemental Figure 2.** Multi-locus sequence analysis dendrogram for *Erysipelothrix* spp, isolates obtained from necropsied southern sea otters (*Enhydra lutris nereis*) (highlighted in red boxes), compared with previously typed isolates from terrestrial mammals, birds, marine mammals, and fish (2). Genes used to assemble the dendrogram include *galk, gpsA, ldhA, prsA, pta, purA, recA,* and *gyrB*.
